# InstaNovo enables diffusion-powered de novo peptide sequencing in large-scale proteomics experiments

**DOI:** 10.1038/s42256-025-01019-5

**Published:** 2025-03-31

**Authors:** Kevin Eloff, Konstantinos Kalogeropoulos, Amandla Mabona, Oliver Morell, Rachel Catzel, Esperanza Rivera-de-Torre, Jakob Berg Jespersen, Wesley Williams, Sam P. B. van Beljouw, Marcin J. Skwark, Andreas Hougaard Laustsen, Stan J. J. Brouns, Anne Ljungars, Erwin M. Schoof, Jeroen Van Goey, Ulrich auf dem Keller, Karim Beguir, Nicolas Lopez Carranza, Timothy P. Jenkins

**Affiliations:** 1 InstaDeep Ltd, London, UK; 2 Department of Biotechnology and Biomedicine, Technical University of Denmark, Kongens Lyngby, Denmark; 3 Novo Nordisk Foundation Center for Biosustainability, Technical University of Denmark, Kongens Lyngby, Denmark; 4 Department of Bionanoscience, Delft University of Technology, Delft, Netherlands; 5 Kavli Institute of Nanoscience, Delft, Netherlands

## Abstract

Mass spectrometry-based proteomics focuses on identifying the peptide that generates a tandem mass spectrum. Traditional methods rely on protein databases but are often limited or inapplicable in certain contexts. De novo peptide sequencing, which assigns peptide sequences to spectra without prior information, is valuable for diverse biological applications; however, owing to a lack of accuracy, it remains challenging to apply. Here we introduce InstaNovo, a transformer model that translates fragment ion peaks into peptide sequences. We demonstrate that InstaNovo outperforms state-of-the-art methods and showcase its utility in several applications. We also introduce InstaNovo+, a diffusion model that improves performance through iterative refinement of predicted sequences. Using these models, we achieve improved therapeutic sequencing coverage, discover novel peptides and detect unreported organisms in diverse datasets, thereby expanding the scope and detection rate of proteomics searches. Our models unlock opportunities across domains such as direct protein sequencing, immunopeptidomics and exploration of the dark proteome.

Mass spectrometry (MS)-based proteomics has revolutionized the way we study proteins on a large scale^1^. Bottom-up proteomics, the main workflow used for system-wide proteomics experiments, relies on the identification of peptides by comparing recorded tandem mass (MS/MS) spectra containing fragment ions with theoretical peptide fragmentation spectra generated from in silico digestion of a protein database^2–4^. At present, the strategy of database search with target-decoy false discovery rate (FDR) estimation is almost exclusively used for both spectrum-centric and peptide-centric acquisition methods^5,6^. The database search approach allows for peptide scoring against acquired spectra and calculation of the FDR of the resulting peptide-spectrum matches (PSMs), which are also strictly controlled at the peptide and protein grouping level^7–9^. Although database search with target-decoy FDR estimation presents a convenient and proven way to reduce the computational search space and control FDR in MS-based proteomics, this approach has critical shortcomings^10,11^. Naturally, a database search narrows the scope of the recorded raw data, and only yields identifications for protein sequences present in the supplied database. Therefore, the selection of the employed database is of great importance, and a poor choice of database can hinder identification of protein isoforms, alternative splicing events, coding single-nucleotide polymorphisms or elucidation of proteins from other organisms not considered for database inclusion. Similarly, database search cannot identify engineered sequences or evolved proteins of interest without knowledge of their sequence, and are agnostic to transcription or translation errors. Another major limitation of database search is the skyrocketing cost in search space complexity and its impact on peptide and protein identification. Inclusion of even a relatively modest number of post-translational modifications (PTMs) exponentially increases the computational cost and processing time of database search^12,13^. This limits searches to only a few PTMs and makes semi-tryptic or open searches—which would allow for the identification of alternative start sites and proteolytically processed proteoforms—time-consuming and computationally expensive^14,15^. The expanded search space also results in an increased false-positive rate, which causes FDR hikes and therefore lower identification numbers^16,17^.

An alternative approach to database search is de novo peptide sequencing, which relies on peptide identification through precursor fragmentation and fragment ion fingerprinting. This approach is the method of choice for bottom-up proteomics when prior sequence information is absent^18,19^. Modern de novo sequencing algorithms have attempted to streamline and automate the process of manual fragment identification and peptide sequencing, achieving impressive results^20,21^. However, such algorithms still suffer from substantial computational costs and high FDRs, rendering de novo sequencing for large-scale experiments unattainable^22,23^. Recently, with the advent of deep learning and powerful neural network architectures, as well as the explosion in MS dataset generation and developments in instrumentation, we are experiencing a renaissance in the field of PSM inference^24–26^, rescoring and de novo sequencing peptide prediction^27–31^. Such approaches hold the promise of accurate peptide identification with linear increases in compute costs for inference, rather than the current exponential cost increases associated with database search. De novo approaches represent a powerful methodology for system-wide sequencing experiments without the need for prior sequence information or additional downsides of database search^32^. By overcoming the limitations of database search, de novo sequencing opens the door to proteomics applications previously considered out of reach. However, so far, such de novo sequencing algorithms have not quite met the performance level required to truly leverage de novo protein sequencing, and their performance compared with database search remains underwhelming.

Here we introduce InstaNovo, a model that exceeds state-of-the-art performance on de novo peptide prediction with substantial increases in precision and recall rates compared with existing tools. InstaNovo is a transformer model that uses multi-scale sinusoidal embeddings^33^ to effectively encode MS peaks. These inputs are processed by nine transformer decoder layers, which cross-attend to the peak embeddings. We apply knapsack beam search decoding for candidate selection and peptide scoring. We also introduce InstaNovo+, an iterative refinement diffusion model inspired by manual human de novo sequencing, which further improves prediction accuracy.

## Results

### Training dataset selection and InstaNovo model architecture

**Fig. 1:**
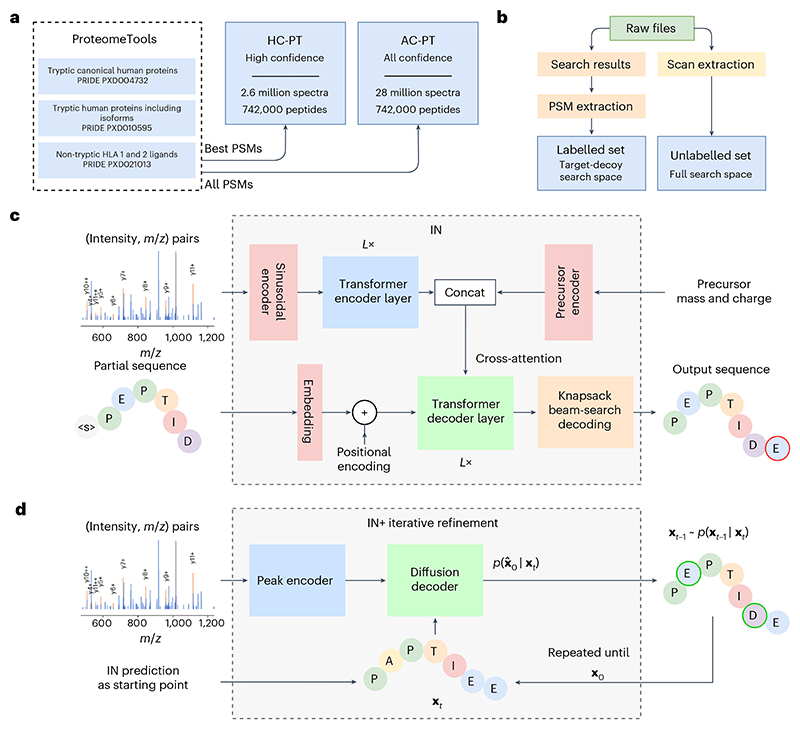
InstaNovo pipeline overview. **a**, ProteomeTools datasets and their PRIDE repository identifiers. Each dataset covers a unique set of synthetic peptides, derived from human protein sequences, which have been measured with MS. **b**, Overview of data extraction and preprocessing steps. Raw data were matched with the results of a database search with target-decoy FDR estimation (controlled at 1%) to create the training dataset of our models. **c**, IN model architecture. The model takes a mass spectrum as input, which is transformed to a latent embedding representation using multi-scale sinusoidal embeddings that encodes the intensity and *m*/*z* vectors. This is passed through *L* transformer encoder layers, each with multiple heads to derive a cross-attention representation of the peaks in the spectrum. Additional precursor information is included and concatenated to form the encoder output, which is cross-attended by *L* decoder layers. The precursor information may alternatively be encoded as the start-of-sequence token in the decoder. The decoder takes in an embedding of the partially decoded peptide sequence, and is responsible for predicting the next residue of the peptide. A knapsack beam search decoding is applied to ensure the model outputs a confident prediction that matches the precursor mass and charge. **d**, Overview over the iterative refinement model, IN+. The model features the IN encoder and a diffusion decoder, which iterates over sequence predictions in a series of timesteps, denoising and refining predictions using a multinomial probability distribution for discrete sequence prediction. *t* is the denoising timestep, **x**_*t*_ is the noised sequence at timestep *t*, **x**_0_ is the denoised sequence where *t* = 0. *p* is the posterior distribution over **x**_*t*−1_ given **x**_*t*_.

Consistent with the literature^34,35^, we reasoned that our model architecture would benefit from training with a large, consistent, well-documented training dataset. Thus, we decided to train our model on the largest available proteomics dataset, the ProteomeTools^36^ dataset (Fig. 1).

Inspired by recent developments in the de novo sequencing field^29,31^, we reasoned that the transformer architecture^37–40^ would be readily adaptable and applicable for de novo peptide sequencing with MS data. This is further supported by work^41^ that builds on transformer-based de novo sequencing models, although there are other architectures that have also shown promising results^42^. We designed our neural network to take the mass spectrum embeddings as model inputs, encoding the intensities and their positions (*m*/*z* in the mass spectrum) in the fragmentation spectra. Recent research has shown that mass spectra vectors can be better represented with multi-scale sinusoidal embeddings^33^. To augment our autoregressive model, we implement knapsack-based beam search decoding, ensuring that the model always outputs a peptide sequence that matches *m*/*z* of the precursor. Together, this architecture constitutes our InstaNovo (IN) model (Fig. 1c and Supplementary Fig. 2a).

### Iterative refinement of predictions improves performance

With recent literature showing diffusion models outperforming previous architectures^43–46^, we reasoned that probabilistic denoising models would be well suited for our spectrum to sequence prediction. In addition, we believed that the iterative refinement properties of denoising models match well with the way humans approach the problem of de novo sequencing, operating with an initial fuzzy prediction based on distinct, unambiguous elements of the spectrum, revisiting and refining the prediction in serial timesteps. On the basis of previous experience^47^, we adapted the denoising principles to suit our purpose, and introduced an iterative refinement model that takes an initial prediction (either random or from the IN model), refines and improves on it by revisiting the information encoded by the spectrum given the updated knowledge provided by the peptide sequence. The model consists of an encoder similar in architecture to IN and a decoder that iteratively refines predictions in 20 steps. The decoder also cross-attends to an embedding of the current timestep, giving the model an indication on how far along the refinement is.

We termed this iterative refinement de novo sequencing model InstaNovo+ (IN+; Fig. 1d and Supplementary Fig. 2b). When the IN predictions were used as the starting input sequences to IN+, we saw a considerable improvement in model performance and recall in our validation sets. This indicates that IN+ is adept in recognizing errors in the initial predictions and correcting them through refinement of the predicted sequences in a series of steps.

### Comparative performance evaluation

**Fig. 2:**
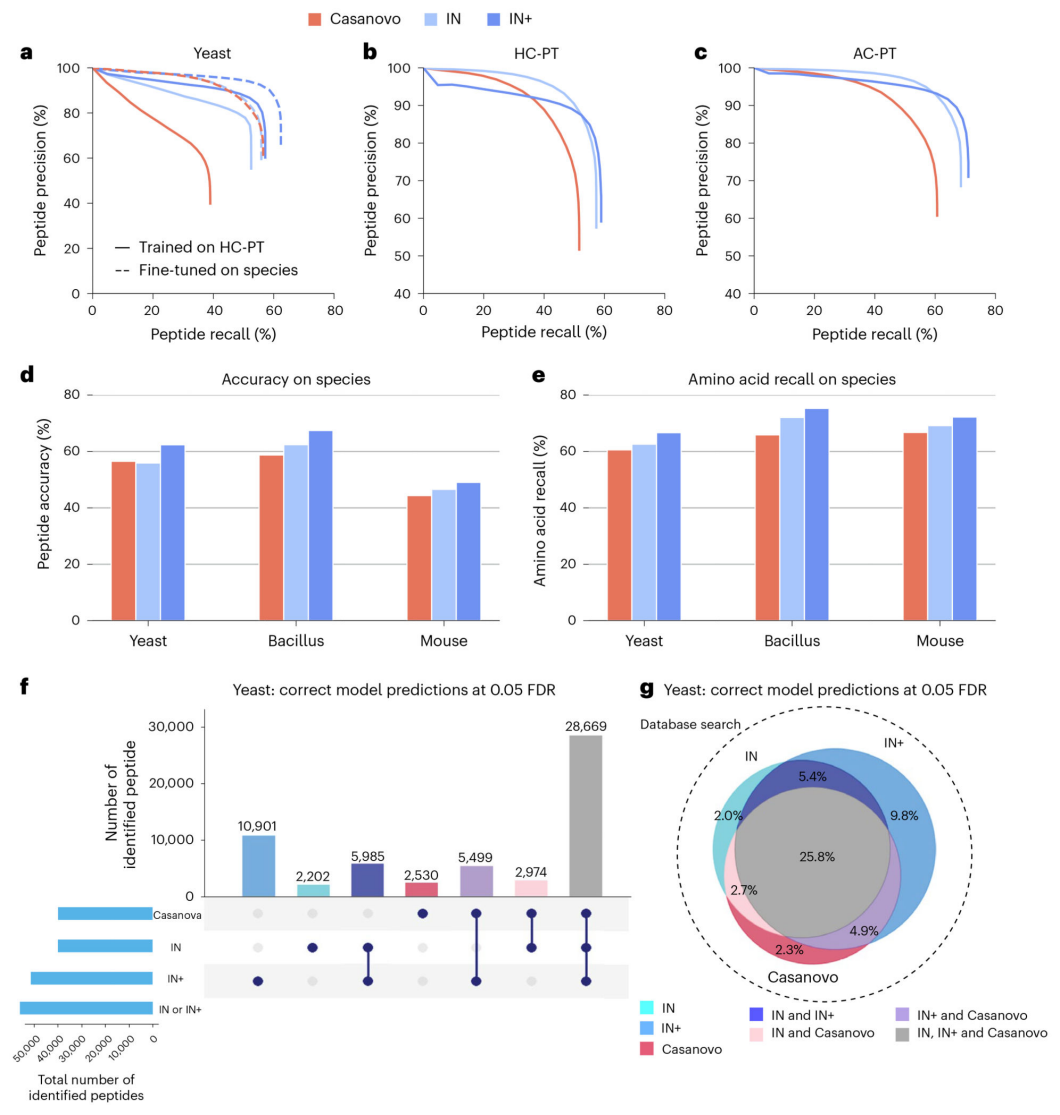
Comparative evaluation of Casanovo, InstaNovo and InstaNovo+. **a**, Peptide-level precision–recall curves on the nine-species dataset, excluding yeast. **b**, Peptide-level precision–recall curves on HC-PT. **c**, Peptide-level precision–recall curves on AC-PT. **d**, Peptide-level accuracy of each model on the high-resolution nine-species dataset, excluding yeast, bacillus and mouse. The model is trained on HC-PT, fine-tuned on the nine-species dataset and then evaluated on the holdout species. **e**, Amino acid-level accuracy of each model on the high-resolution nine-species dataset, excluding yeast, bacillus and mouse. **f**, Peptide-level UpSet plot illustrating the intersection of correct predictions made by the fine-tuned IN, IN+ and Casanovo models on the nine-species dataset, excluding yeast, when evaluated at an FDR of 0.05. **g**, Peptide-level Venn diagram illustrating the same intersections as **f**, but showing them as percentages (recall) of the database search ground-truth (ms_ninespecies_benchmark) dataset, which is illustrated by the area of the circle with the dotted edge. Areas in the Venn diagram are approximate, owing to the imperfection of the Venn algorithm.

We conducted performance evaluation of IN by comparing it with the current state-of-the-art model, Casanovo^29^. This model was selected as it also used a transformer architecture and reported leading-edge performance, making it an ideal benchmark. We used two benchmark datasets: the high-resolution nine-species dataset^30^, which serves as a standard benchmark for evaluating deep learning de novo peptide sequencing tools, and the ProteomeTools^36^ dataset, which provides a more comprehensive collection of high-quality mass spectra derived from synthetic peptides. We implemented PointNovo^48^ but found that it never converged to a comparable level of performance when trained on high-confidence ProteomeTools (HC-PT), and so it was excluded. When we assessed the peptide-level precision–recall curve comparing the models trained only on HC-PT, and those trained on HC-PT and fine-tuned on the nine-species dataset, we see IN+ and IN outperforming Casanovo when trained on HC-PT, whereas Casanovo is comparable with IN when trained on HC-PT and fine-tuned on the nine-species dataset. IN+ outperforms Casanovo and IN when fine-tuned (Fig. 2a). We also evaluated the HC-PT trained models on HC-PT and all-confidence ProteomeTools (AC-PT), respectively (Fig. 2b,c). On HC-PT, the precision–recall curve of IN showed improved calibration compared with IN+, with higher peptide precision for the same recall values. We expect this is due to the way we estimated the lower bound of the diffusion model confidence, which is not as straightforward as autoregressive models. On the nine-species dataset, we evaluated the model accuracy on three species (Fig. 2d,e). We see that IN+ consistently outperforms both Casanovo and IN, for both peptide-level accuracy and amino acid recall. We found that although IN+ in itself marginally improves recall, it ends up predicting not only many of the same peptides as IN but also different ones. As such, IN+ does not merely constitute a refinement in our base model, but can be used in addition to IN, overall substantially increasing the number of peptides predicted with low FDR.

We next used the database search results to ground our search and derive a surrogate confidence threshold for FDR estimation. Comparing the PSMs identified in database search with model predictions, we calculated the confidence threshold of the de novo peptide sequencing models that can yield the predictions with 5% FDR. We evaluated the predictions above this confidence threshold that are identical to the database search PSMs. In the nine-species yeast dataset, a database search identified 111,312 PSMs after filtering of a maximum peptide length of 30 and a maximum of 800 peaks in the spectrum. Within that PSM pool, we found that Casanovo predicted 39,659 PSMs at 5% FDR with 2,530 not found in either IN or IN+; IN predicted 39,830 PSMs (2,202 unique) and IN+ identified 52,633 PSMs (10,901 unique), 32.71% more than Casanovo. Together IN and IN+ identified 56,230 PSMs, 41.78% more than Casanovo, which constituted a substantially improved performance of both models when combined (Fig. 2f,g). This trend still held true for the other two datasets (HC-PT and AC-PT), although the improvement was smallest for HC-PT (Extended Data Fig. 2a–d). Error analysis indicated that IN and IN+ are incorrectly classifying predictions in the same categories as Casanovo (Extended Data Fig. 3).

### InstaNovo adds value and robustness to bottom-up proteomics

**Fig. 3:**
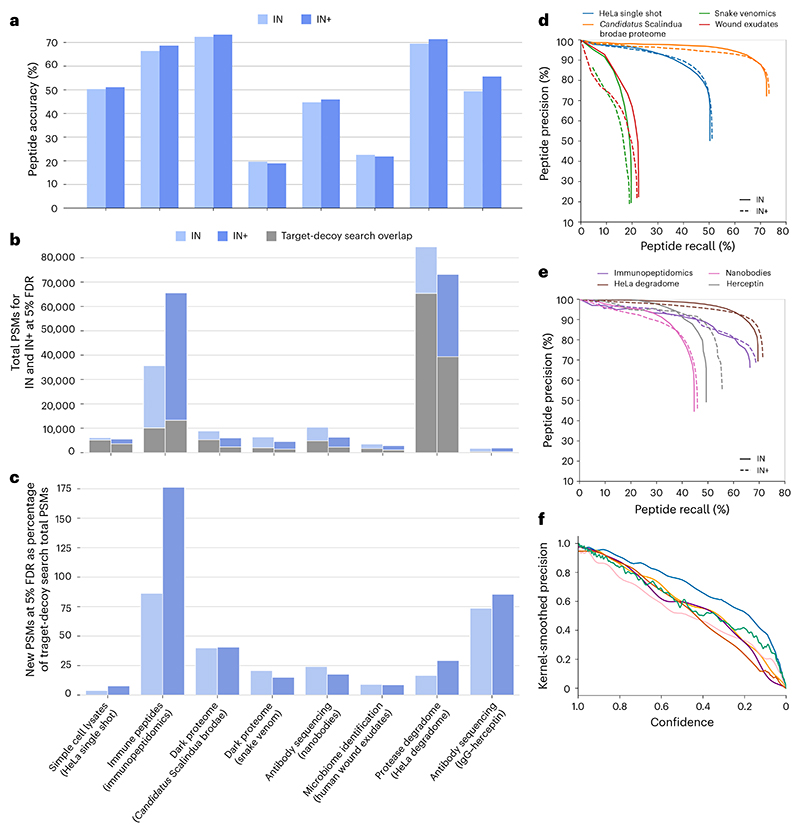
Performance of InstaNovo and InstaNovo+ on the labelled application-focused datasets. **a**, Peptide-level accuracy of IN and IN+ on each application-focused dataset. **b**, Total number of PSMs for IN and IN+ models at 5% FDR. Overlap with database search PSMs is shown in grey. **c**, Novel PSMs at 5% FDR for IN and IN+, expressed as a percentage of database search total PSMs. **d**, Peptide-level precision–recall curves for proteomes explored in this study. These consist of HeLa cell lysate proteome, ‘*Candidatus* Scalindua brodae’ proteome from a co-enrichment culture, snake venom proteomes and the proteome from human patient wound exudates as extracted from dressings. **e**, Comparison of peptide-level precision–recall curves for both models on the datasets where novel sequences were involved. These were HLA peptide-enriched samples, nanobodies and the antibody herceptin, as well as a HeLa proteome dataset including semi-tryptic and open search peptides. **f**, Kernel-smoothed precision of model confidence distributions across multiple datasets for IN.

We evaluated IN and IN+ on eight validation datasets within major areas of interest, that is, including simple cell lysates (HeLa single shot), immune peptide identification (immunopeptidomics), the dark proteome (‘*Candidatus* Scalindua brodae’; snake venoms), antibody sequencing (nanobodies; IgG–herceptin), microbiome identification (human wound exudates) and the protease degradome (HeLa degradome). Database search was applied to each, with the search results and number of spectra outlined in Extended Data Table 1. In a given dataset, IN achieved up to 72.4% peptide accuracy and IN+ achieved up to 73.6% peptide accuracy (‘*Candidatus* Scalindua brodae’ proteome) without further fine-tuning on individual datasets, and only including the training evaluation rounds. The performance fluctuated depending on the dataset, resulting in an average of 48.3% peptide accuracy ± 19.4% s.d. for IN, and 51.5% peptide accuracy ± 21.1% s.d. for IN+ on these 8 biological application-oriented datasets (Fig. 3a and Extended Data Table 2). At 5% FDR, IN predicts a median of 4,014 PSMs (Fig. 3b), or an average of 34% novel PSMs at 5% FDR compared with the total PSMs in database search results (Fig. 3c). Within the database search results, IN+ finds on average 3% more PSMs that were not covered by IN, while improving peptide accuracy by 1.5% on average (Extended Data Table 3). Precision–recall curves in application-focused datasets show considerable variance depending on sample type and origin (Fig. 3d,e), while model precision as a function of confidence is generally conserved, especially for confidence values above 95%, with the exception of the snake venom proteomics and the nanobodies dataset (Fig. 3f).

### Additional evaluations on application-focused datasets

We further performed in depth characterization of the eight application-focused datasets to gain a deeper understanding of the biological insights gained by IN and IN+ analysis. Additional details can be found in Supplementary Note 9.

### InstaNovo detects more than half of the human proteome from HeLa cells and expands the sequence coverage of novel biologics

**Fig. 4:**
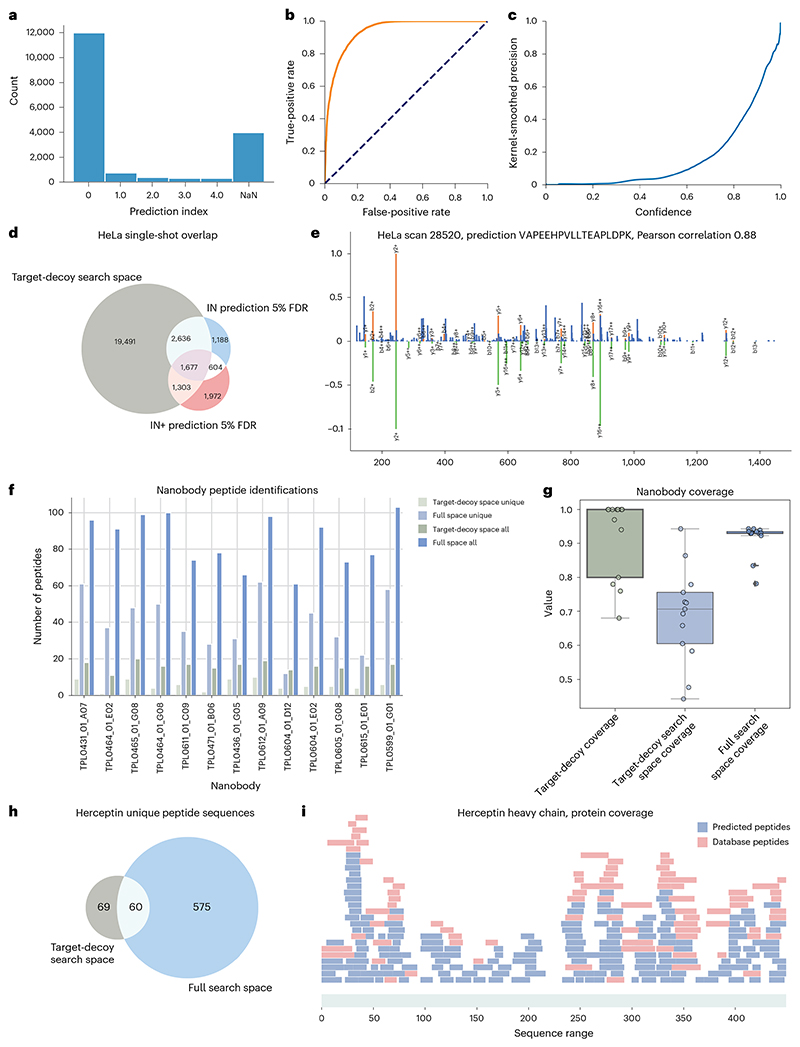
InstaNovo achieves good accuracy on the established HeLa proteome and sequences therapeutics in different formats. **a**, Barplot of prediction distribution index with the highest confidence matching the precursor mass. NaN, not a number. **b**, Receiver operating characteristic (ROC) curve analysis for HeLa single-shot proteome IN predictions. Orange line: sensitivity as a function of false positive rate. Dashed line: true positive and false positive parity. **c**, IN+ prediction confidence in the HeLa single-shot proteome. **d**, IN and IN+ predictions and their overlap with database search PSMs at 5% FDR in the HeLa single-shot proteome. **e**, Mirror plot of experimental spectrum (top) and Prosit predicted spectrum (bottom), in a prediction sequence showing better correlation than the database search PSM. **f**, Barplot of total and unique peptides for the nanobodies analysed. **g**, Sequencing coverage for nanobodies (*n* = 13, median as centre line, 25th to 75th percentiles as bounds of the box, whiskers extending to 1.5 times the interquartile range from the bounds of the box, with minima and maxima beyond the whiskers plotted as individual points) analysed for database search, IN-predicted database search and IN-predicted full search at 5% FDR. **h**, Venn diagram for peptides sequences matching to herceptin in the six protease digests analysed with database search and IN predicted in the full search space. **i**, PSMs for database search results and IN-predicted peptides for the herceptin heavy chain.

First, we conducted a benchmark study on the lysate of HeLa cells. The results from this study (Fig. 4a–e and Extended Data Fig. 4) suggested that IN generates high-confidence predictions that support and expand database search results even in the most comprehensively characterized proteomes. IN was able to achieve 49.6% recall in the HeLa single-shot dataset, assigning correct (identical to the database search) sequences for 8,774 PSMs. Using a confidence cut-off equivalent to 5% FDR for sequence predictions, IN increased the database search PSM identification rate by 7.5%, identifying 1,338 more PSMs in the MS/MS scans that did not result in any database search hits.

Next, we investigated our model’s performance in de novo sequencing of novel, engineered biomolecules (see Supplementary Note 9 for preparation details). Notably, we sequenced 13 nanobodies and obtained 7,536 matches mapping to 613 peptides when expanding the search to the full search space (all MS/MS spectra) of our runs, which presented a 6-fold peptide detection increase compared with the PSM space from database searches (Fig. 4h). The unique peptide sequences detected for a given nanobody increased from 5 to 40, a striking 8-fold increase in average unique sequences when contrasted with the database search space. We also applied our model to a publicly available dataset evaluating MS-based antibody sequencing^49^, where the authors used nine different proteases and two fragmentation activation types to sequence herceptin. Importantly, it increases protein coverage to 92.87% and 100% for heavy and light chains, respectively (Fig. 4i). The results from this study (Fig. 4f–i and Extended Data Fig. 6) indicated that our models are adept at novel protein sequencing with IN and IN+ matching database results, while simplifying the sequencing workflow.

### InstaNovo finds novel proteins and pathogens in proteomes

**Fig. 5:**
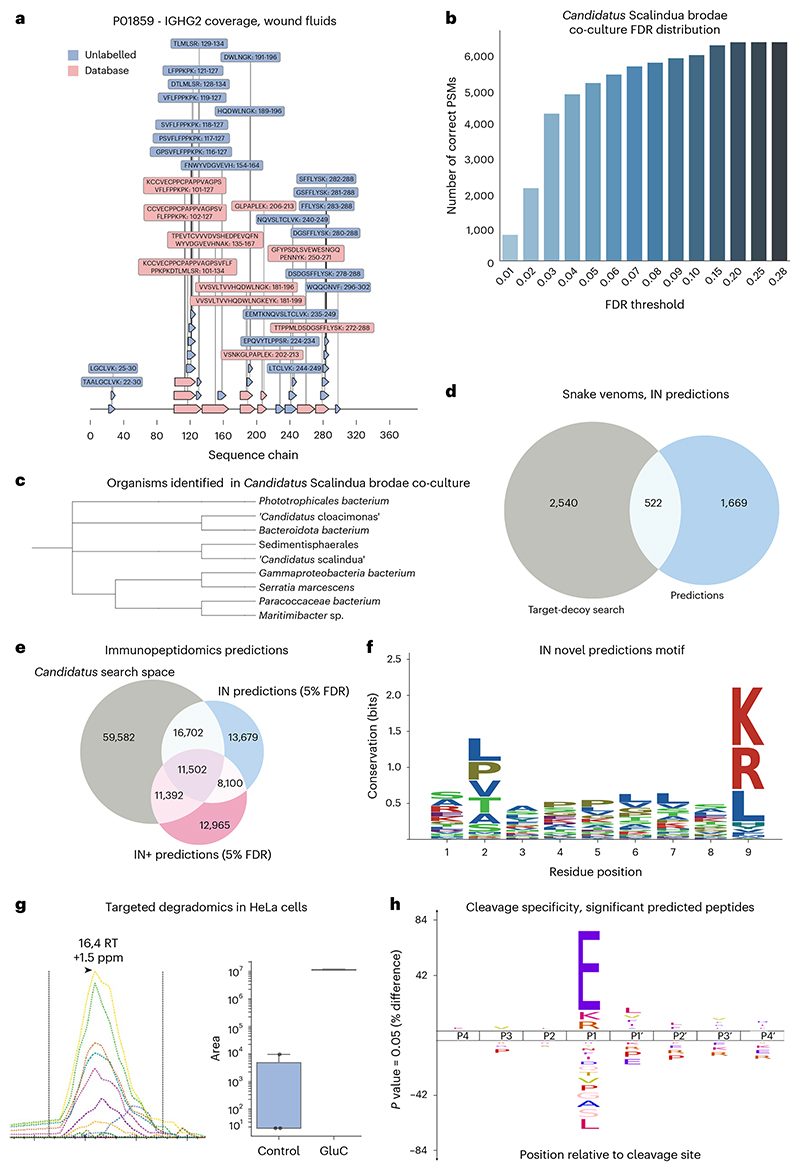
InstaNovo increases protein coverage, identifies novel organisms, and detects semi- and non-tryptic peptides. **a**, Protein coverage and peptide sequences for UniProt ID P01859 - IGHG2 (immunoglobulin heavy constant gamma 2 chain) in human wound fluids, where database search peptides and novel predictions with IN are shown. **b**, Correct PSMs for different precision thresholds in the ‘*Candidatus* Scalindua brodae’ proteome. **c**, Phylogenetic tree of a representative sample of additional organisms identified in the co-culture. **d**, Venn diagram of database search and novel IN predictions of peptide sequences at 5% FDR from snake venom proteomics that map to the proteomes database used. **e**, Venn diagram of database search, IN and IN+ predictions at 5% FDR peptide sequences matching the proteome database used from immunopeptidomics dataset. **f**, Shannon information content of residues in sequence positions of immunopeptidomics experiments. **g**, PRM monitoring of fully GluC-generated peptide ATVWIHGDNEENKE, and its abundance in the two conditions (*n* = 3, median as centre line, 25th to 75th percentiles as bounds of the box, whiskers extending to 1.5 times the interquartile range from the bounds of the box, with minima and maxima beyond the whiskers plotted as individual points). RT, retention time in minutes. **h**, GluC specificity profile from statistically significant predicted PSMs matching database search results.

Following the above results, we questioned how our model would perform in complex samples where the presence of multiple organisms is suspected. For that, we utilized wound fluid exudates from human patients with venous leg ulcer^50^. We extended albumin mapping to 1,225 PSMs with 254 unique peptides (most semi- or non-tryptic), a 10-fold increase compared with the database search space, and observed analogous results in other proteins (Fig. 5a). Importantly, we mapped unique sequences to 5 of *Pseudomonas aeruginosa*, 23 of *Escherichia coli* and 24 of *Citrobacter* sp. proteins, with a substantial number of sequences mapping to multiple proteomes. We validated the presence of *E. coli* and *P. aeruginosa* in both wound exudates by PCR of the 16S rRNA gene for these organisms (Extended Data Fig. 5).

We next looked into how IN performs in the field of metaproteomics. We chose a co-culture of an enrichment reactor for the marine bacterium ‘*Candidatus* Scalindua brodae’. We examined the 1,937 sequences that did not map to our protein databases by comparing them with sequences in genome databases. This revealed potential additional species present in our samples, such as *Phototrophicales bacterium*, ‘*Candidatus* Scalindua arabica’, *Phycisphaerales bacterium, Bacteroidota bacterium* and *Gemmatimonadota bacterium* (Fig. 5b,c). Our results demonstrate that IN is suitable for metaproteomics applications, with no prior knowledge about presence of these organisms required. Furthermore, we investigated the application of our models to samples where limited genomic information is available. We therefore picked a dataset that recently described the proteome composition of 26 medically relevant snake venoms from sub-Saharan Africa^51^, arguing that as not all genomes are available and these proteomes were searched against a pan-snake proteome database, we might detect potential novel sequences unique for some of these species. For example, ‘SLGGVTTEDCPDGQNLCFK’ aligned with the isoform 1 sequence of MTLP-2 from *Naja kaouthia*, a snake species that was absent from our input dataset. Overall, these results (Fig. 5d) indicated that there were novel hits with undetected, or not included in the database, search sequences. These could provide insights into novel proteins, isoforms or single-nucleotide polymorphisms in these samples.

### InstaNovo identifies peptides in immunopeptidome and degradome

Subsequently, we asked whether our de novo sequencing models could be applied to the sequencing of human leukocyte antigens (HLA) peptides for the analysis of immunopeptidomics experiments. Remarkably, IN predicts 3,495 novel peptides compared with the target-decoy search, increasing the peptide identification rate by 41.53%. IN+ at 5% FDR detected 11,392 more PSMs from the target-decoy search and predicted 12,965 novel PSMs (Fig. 5e). The 9-mer peptides identified with IN showed a motif consistent with major histocompatibility complex bound peptides, exhibiting preferences for certain residues in positions 2 and 9, supporting the model predictions (Fig. 5f). These results indicated that IN performs well in open searches, is adept in prediction of HLA peptide sequences and can substantially enhance identification rates in immunopeptidome datasets. Finally, we questioned our model’s performance in limited processing or degradomic samples, where proteolytic substrates and their discovery are of interest. We prepared and applied our model to a HeLa proteome incubated with the protease GluC. IN predicted 4,635 new peptide sequences and improved the peptide detection rate by 11.29% (Extended Data Fig. 7a,b). Importantly, IN predicted 1,222 new sequences that match the protease profile, that is, are preceded by glutamate residue in the respective protein sequences these peptides map to (Extended Data Fig. 7c,d). Subsequently, we wondered whether these cleavages reflected bona fide peptide detections that were missed by database searches. We were able to identify several high-confidence, semi-tryptic or fully GluC-generated peptides with targeted proteomics. We monitored their fragmentation transitions in both conditions (Fig. 5g), and obtained a specificity profile with glutamate before the cleavage site significantly over-represented in statistically significant peptides (Fig. 5h). The results from this study confirmed our hypothesis that IN can be applied to the detection of protease substrates at a system-wide scale.

## Discussion

By expanding the scope of proteomic applications and providing insights into previously inaccessible protein landscapes, de novo peptide sequencing is a promising tool for advancing our understanding of a wide range of complex biological systems. Here we introduce the IN and IN+ models and analyse their predictive performance in several application domains, including the sequencing of engineered biomolecules, immunopeptidomics and exploration of the dark proteome. We demonstrate improvements in peptide searches and computational costs, and benchmark against another tool used for de novo sequencing, Casanovo. To our knowledge, these results represent a notable improvement over other algorithms for de novo sequencing in bottom-up proteomics and constitute a promising step in replacing or complementing database searches.

Beyond the general improvements over state-of-the-art de novo peptide sequencing tools, we present applications of our model in several questions in biology. We uncover novel biological findings across eight different datasets, including the identification of proteins in HeLa cells undetected by database search, the expansion of the immunopeptidomics dataset by 175% more peptides and the characterization of novel proteolytic cleavages. Given our results and the diversity of the datasets explored in this study, we expect that the model may generalize with high accuracy and satisfactory performance across organisms and biological samples. We anticipate future applications of the model in several other research areas, such as proteogenomics^52^, gut microbiome studies^53^ and studies aiming to explore unreported proteoforms^54^. We also hope that our models find suitable applications in the emerging field of single-cell proteomics, where increasing PSM detection rates from minute sample amounts is of paramount importance^55,56^.

We expect that by fine-tuning our models on specific tasks, such as big datasets or individual PTMs, they will learn to recognize novel natural or induced chemical modifications of peptide sequences, expanding its applications in chemoproteomics, PTM detection and discovery, as well as multiplexed proteomics. We also expect our models to generalize well to lower-resolution spectra and various fragmentation techniques. However, further research is needed to assess the performance and generalization of IN and IN+ in different types of mass spectrometer (for example, instruments with time-of-flight or ion trap detectors), different resolution of MS/MS scans and their effect in performance and prediction confidence, as well as different fragmentation techniques for PTM discovery. We await investigation of different acquisition schemes, such as data-independent acquisition, and model input adaptation by the creation of pseudo-MS2 spectra^57,58^, facilitating higher detection rates even for applications requiring very high sensitivity.

Following recent trends^59,60^, we anticipate hybrid searches with multiple orthogonal methods of PSM predictions, downstream rescoring algorithms and ensemble models to be increasingly useful in utilizing the full recorded spectrum space and maximize detection rates. It has to be noted that in our characterization and evaluation of the model, we consider database search PSMs as the ground truth for peptide detection in our dataset. This assumption might be flawed, as database search space PSMs and confidences might be incorrect or incomplete. We believe that our models can efficiently be used to corroborate, correct and/or disprove database search PSMs, increasing detection rates and improving peptide prediction precision. We also speculate that comprehensive post-processing evaluation of model predictions and multivariate filtering based on peptide features and spectrum similarity will increase the sensitivity and fidelity of PSMs. Post-processing filters could also serve as a funnel for refinement of predictions with our IN+ model, further leveraging the iterative refinement of predictions with diffusion, which currently is only scratching the surface of its potential. We further believe that our models perform adequately well in prediction of non-tryptic peptides, especially if fine-tuned to allow for the use of different peptidases for proteolysis and thereby increasing protein coverage and sequencing. We predict that deep learning approaches will be critical in overcoming the complexity of database searches, and we expect reduced search times for ultrafast sequence predictions in digestion-agnostic proteomics searches.

Together, our results and those of others show that scale is the most determining factor in de novo peptide sequencing model performance, as with other fields where the transformer architecture was employed^35^. We expect to further increase model performance by taking advantage of the vast amount of MS datasets available in repositories. We also anticipate widespread adoption by peers, and look forward to further exploration of fine-tuning, protein inference and assembly, as well as building applications on top of our base model for hybrid or de novo searches.

## Methods

### Data

#### Training dataset retrieval and preparation

**Extended Data Fig. 1:**
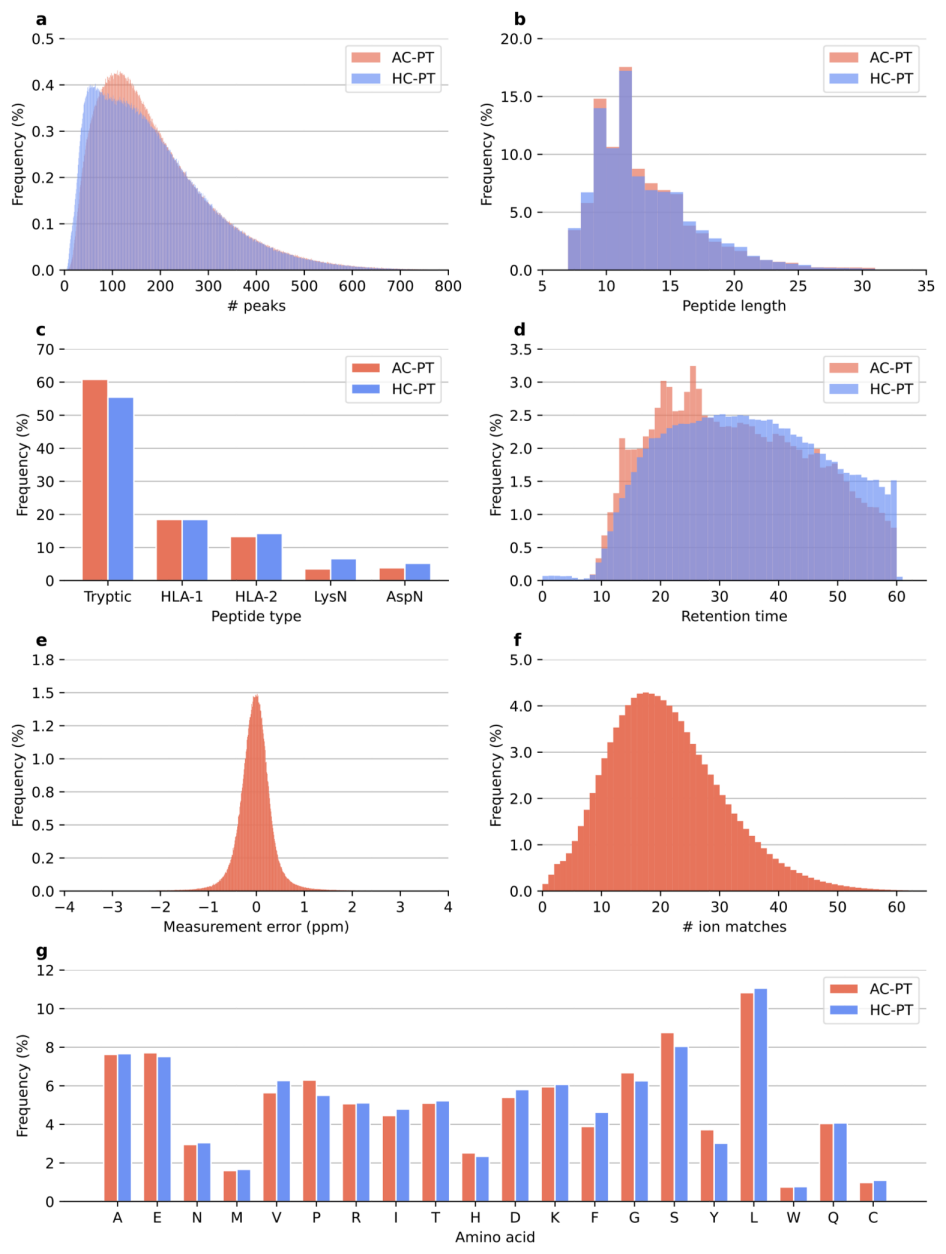
ProteomeTools descriptive statistics for all-confidence PSMs (AC-PT) and high-confidence PSMs (HC-PT). **a**, Number of peaks per spectrum. **b**, Peptide length of PSM sequences. **c**, Distribution by peptide type, including tryptic, HLA-I, HLA-II, LysN, and AspN. **d**, Retention time distribution. **e**, Distribution of measurement error (ppm) in AC-PT. **f**, Ion matches for PSM scans distribution in AC-PT. **g**, Amino acid frequency in PSM sequences.

IN was trained on the large-scale ProteomeTools^36^ dataset, which has been recorded with modern, state-of-the-art instrumentation, containing high-resolution spectra for peptides of human origin. This dataset comprises over 700,000 synthetic tryptic peptides covering the entirety of canonical human proteins and isoforms, as well as encompassing peptides generated from alternative proteases and HLA peptides. We used the data from the first three parts of the ProteomeTools project, and split the database search results into two datasets. The first dataset is derived from the evidence results of the MaxQuant^61^ searches available in the repository, and contains the highest-confidence PSMs per peptide and is therefore referred to as the HC-PT dataset. The second dataset contains all PSMs regardless of quality (derived from the MS results of the searches), and is referred to as the AC-PT dataset. The HC-PT dataset contains 2.6 million unique spectra, and the unfiltered AC-PT dataset contains 28 million total spectra. Both datasets contain 742,000 unique peptides (Fig. 1a). Distributions of the dataset properties show expected behaviour in terms of *m*/*z*, charge, measurement error and so on (Extended Data Fig. 1). After obtaining the training data from the repository, we devised a pipeline to extract the spectrum information and associated metadata we believed were needed for model training (Fig. 1b and Supplementary Fig. 1).

In more detail, to ensure a consistent analysis, only the 3x high-energy collision-induced dissociation (HCD) data were utilized, as they provided an inclusion list and employed 3 different HCD fragmentation energies. The raw data files were converted to mzML format using the Proteowizard MSConvert tool^62^, with default settings. The result files obtained from MaxQuant^61^ (‘evidence.txt’ or ‘msms.txt’ for high-confidence or full dataset, respectively) were employed to extract scan indices for identified peptides, as well as the associated metadata (precursor mass, charge, measurement error, retention time) for each PSM. To facilitate further analysis, the pyOpenMS Python^63^ wrapper of the OpenMS C library was utilized. This tool enabled the reading of mzML files, extraction of scans and association of the scans with the PSM metadata. To refine the dataset and set a padding threshold for the model input features, PSMs were filtered based on specific criteria. Only peptides with a length of 30 or fewer residues and a maximum of 800 peaks in the spectrum were included in the analysis. In all of our experiments, we used residues with the following PTMs: carbamidomethylation for cysteine, oxidation for methionine, and deamidation for asparagine and glutamine.

### Data splits

We did a 80:10:10 train/validation/test split for HC-PT and AC-PT based on the unique peptide sequences. When splitting, we ensured that there was no leakage between the HC-PT sets and the AC-PT sets (that is, no HC-PT train samples are present in the AC-PT test set, and so on). All models and hyperparameters were chosen based on their validation set performance. Test-set results were computed only when writing up the paper and used for the reported figures. All results shown in the paper are reported on the test set. For yeast, bacillus and mouse, we used the splits as defined in DeepNovo^30^ and PointNovo^48^.

### Model implementations

#### Development of InstaNovo architecture

The IN architecture is based on the transformer encoder–decoder architecture^64^. Similar to Point-Novo^48^ and Casanovo^29^, we represent our MS2 spectra as the set of *N* peaks (**m, I**), where **m** = *m*_1_, *m*_2_, …, *m*_*N*_ and **I** = *I*_1_, *I*_2_, …, *I*_*N*_ represent the sets of *m*/*z* and intensity, respectively. To encode these peaks, we employ multi-scale sinusoidal embeddings^33^. We process these encoded peaks through a transformer encoder layer, allowing the model to self-attend and extract relative information between the peaks. The encoder output is concatenated with a learnt latent spectrum and a representation of the encoding of the precursor. The precursor mass *m*_prec_ and charge *c*_prec_ are encoded with a sinusoidal encoding and embedding layer, respectively, after which they are summed to represent the precursor embedding. This precursor may alternatively be encoded as the start-of-sequence token in the decoder, but we found no difference to model performance. The encoder has 9 layers, each with 16 heads, a hidden dimension of 768, and a feed-forward dimension of 1,024. This encoder allows the fragment ions and their intensities to self-attend to other ions present in the spectrum.

The transformer decoder, also consisting of 9 layers with 16 heads each, makes use of causal autoregressive decoding. This enables the model to take in the previous residues from the predicted sequence and autoregressively predict the next token. The partially decoded sequence is encoded through an embedding layer and a standard sinusoidal positional encoding is added. The input sequence is automatically prepended with a start-of-sequence token. The decoder cross-attends over the encoder output, latent spectra and precursor encoding.

For the causal autoregressive decoding, we implement knapsack beam search decoding. This eliminates the need for multiple predictions and retains performance while increasing model confidence and decreasing FDRs in the full search space. IN recall is marginally reduced across datasets (0.05–0.2%) compared with a standard beam search with 5 predictions per spectrum, and peptide inference takes longer compared with beam search, but reductions in almost all error types justify its use.

IN has 95 million parameters in total. To train IN, we implement the model in PyTorch^65^, with PyTorch Lightning^66^ being used to handle the training loop. The loss function computes the cross-entropy between the predicted model logits and the ground-truth peptide. All training and model hyperparameters are provided in Supplementary Table 1.

#### Iterative refinement with InstaNovo+

After our initial model training and promising results in sequence decoding, we speculated that next-token prediction is not the most optimal approach to mass spectrum sequence decoding.

Under HCD and collision-induced dissociation fragmentation, the most intense ions are the b and y ions^67–70^ of the peptide, with the y ions of tryptic peptides generally having better readout properties, potentially due to charge localization. For that reason, many de novo sequencing models start token prediction from the right-hand side of the sequence, as we also do for our base model IN. However, we argued that as internal y or even b ions are more intense, there might be an advantage in exploring approaches that decode the peptide sequence all at once instead of performing next-token prediction (Supplementary Fig. 5).

Hence, in addition to IN, we introduce IN+, based on a similar transformer architecture but with a different goal. Rather than autoregressive decoding, the IN+ model is trained to perform multinomial diffusion^47,71^. This means the model is trained to iteratively remove noise from a corrupted sequence (see Supplementary Note 2 for further details). The full model architecture is given in Supplementary Fig. 2b.

When decoding IN+, we decode five samples for each spectrum. The sequence that matches the precursor mass with the highest log probability under the model is selected as the IN+ prediction. In the case where we start with an IN prediction and none of the IN+ predictions satisfy the precursor mass, we instead fall back to the IN prediction used at *t* = 15 (which should always fit the precursor).

### Metrics and benchmarks

We use peptide recall as our main benchmarking metric for testing and validation datasets. As this is the more stringent of metrics used in de novo sequencing algorithm evaluation, we believe that this metric reflects our model’s performance the best. We also report peptide precision, as well as amino acid residue precision, recall and error rates for our training and validation datasets. We formulate our metrics as done in ref. 49 (see Supplementary Note 4 for details). We further compared our models with baselines using the entire receiver operating characteristic curve rather than just the precision and recall at a single confidence threshold. We obtained these by varying the confidence threshold from the highest to the lowest values obtained in an evaluation dataset and plotting the resulting pairs of (amino acid or peptide level) precisions and recall values.

We decoded peptides from our models using beam search with knapsack filtering (Supplementary Note 5, Algorithm 1). This ensured that the system always found a peptide that fit the precursor mass, improving overall performance and reducing the frequency of almost all individual error types. Beam search (with beam width *B*) is a variant of breadth-first search where at each step, the frontier is pruned to the *B* highest scoring sequences. We use knapsack filtering in beam search to allow only amino acid sequences that can be continued so that their theoretical mass matches the precursor mass to a 50 ppm relative difference. See Supplementary Note 5 for further details.

### Application-oriented datasets

#### Nanobodies

The nanobodies included in this study (Supplementary Table 2) were discovered using phage display technology (see Supplementary Note 9 for further details). The nanobody concentration was determined by measuring the absorbance at 280 nm in a NanoDrop One (ThermoFisher Scientific). From each stock solution, 10 μg of nanobody was transferred, the buffer was exchanged and the volume was reduced with SP3 bead clean-up^72^ and following on-bead digestion. In brief, pure ethanol was added to a final concentration of 80%. Fifty micrograms of each hydrophobic and hydrophilic beads (Cytiva, Sera-Mag Carboxylate-Modified [E7] Magnetic Particles 24152105050250 and Sera-Mag SpeedBead Carboxylate-Modified [E3] Magnetic Particles 65152105050250) were added to the solution, and incubated in a thermomixer at room temperature, at 800 rpm, for 15 min to allow binding. Samples were placed in a magnetic rack and the solvent was removed. The remaining beads and bound proteins were washed 3 times with 90% ethanol, and were finally resuspended in 20 μl of 2.5 M guanidine hydrochloride (GuHCl; G3272 Sigma-Aldrich) and 250 mM HEPES solution (4-(2-hydroxyethyl)piperazine-1-ethanesulfonic acid; 7365-45-9 Sigma-Aldrich). Nanobodies were reduced and alkylated with 10 mM TCEP (tris(3-hydroxypropyl triazolyl methyl)amine; 762342 Sigma-Aldrich) and 40 mM CAA (2-chloroacetamide; 79-07-2 Sigma-Aldrich), incubated for 10 min at 95 °C. Samples were diluted 5 times in MilliQ water, and 200 ng trypsin (V5280 Promega Gold) was added to a 1:50 protease:proteome ratio, assuming no losses. Samples were digested overnight, at 37 °C, 450 rpm. The next day, samples were placed on a magnetic rack and the solution was transferred to a new tube. Approximately 500 ng of peptides, assuming no losses, was acidified and loaded on EvoTips with the standard loading protocol^73^ for MS analysis. The samples were analysed using the EvoSep One liquid chromatography platform, in line with an Orbitrap Exploris 480 mass spectrometer equipped with a FAIMSpro device.

Peptides were separated with a PepSep C18 column (15 cm × 75 μm, 1.9 μm PepSep, 1893473), over 31 min, employing the Whisper100 40SPD method. Peptides were ionized with nanospray ionization with a 10 μm emitter (PepSep, 1893527), and spray voltage of 2,300 V in positive-ion mode, and ion transfer tube of 240 °C. The total carrier gas flow was set to 3.6 l min^−1^, and FAIMS was operated at standard acquisition. Spectra were acquired in data-dependent resolution mode, under two different compensation voltages of −50 and −70 V, with identical settings. The cycle time was set to 2 s, with MS1 spectra acquired with 60,000 resolution, a scan range of 375–1,500, a normalized AGC target of 300%, a radio-frequency lens of 40% and an automatic injection time. Filters were set for peptide MIPS mode, inclusion of charge states 2–6, dynamic exclusion of 60 s with 10 ppm tolerance and an intensity threshold of 10,000. MS2 spectra were acquired with an isolation window of 1.6 *m*/*z*, normalized HCD of 30%, Orbitrap resolution of 30,000, first mass at 120 *m*/*z*, normalized AGC target of 100% and an automatic injection time. Data analysis was performed in Proteome Discoverer^74^ v2.4, with Sequest HT^75^ as the search engine. The database used was the *E. coli* reference proteome (Uniprot reviewed, UP000284592, 4,360 sequences, accessed 1 December 2022) concatenated with the nano-body sequences, and additional dynamic modifications of acetylation or methionine loss at the protein N-terminus, along with methionine oxidation, and static modification of carbamidomethylation. FDR control was performed with Percolator, at 1% and 5% target FDRs. Precursor quantification was performed with the Minora Feature Detector and Feature Mapper nodes in the processing and consensus workflows, respectively. Abundances were based on unique and razor peptides and above a signal-to-noise ratio of 5, and normalized based on total protein amount. PSMs at 1% FDR were exported for further processing, data extraction and model validation.

#### HeLa proteome

HeLa cells were cultured in T25 flasks with Dulbecco’s modified Eagle medium (10565018, ThermoFisher Scientific) until confluency. Cells were pelleted with centrifugation, and resuspended in 6 M GuHCl. Proteins were reduced, alkylated and digested as for nanobodies above, with an additional LysC digestion for 1 h at 1:100 protease:protein ratio, before tryptic digestion. Two-hundred nanograms of peptides, assuming no losses, were acidified and analysed with a nLC E1200 in line with an Orbitrap Exploris 480 mass spectrometer equipped with a FAIMSpro device. Peptides were separated with an 15 cm × 75 μm, 2 μm EASY-SpayTM column (ThermoFisher Scientific, ES904) over a 70 min gradient, starting at 6% buffer B (80% acetonitrile, 0.1% formic acid), increasing to 23% for 43 min, then to 38% for 12 min, 60% for 5 min, 95% for 3 min, and staying at 95% for 7 min. Peptides were ionized with electrospray ionization with a positive-ion spray voltage of 2,000 V, and ion transfer tube of 275 °C. The rest of the method settings were as described above, with the difference of top-20 data-dependent scans, and normalized HCD of 28% for MS2 spectrum acquisition. Data analysis was performed as above, with the only differences being the use of human database (Uniprot reviewed, UP000005640, 20,518 sequences, accessed 5 March 2023), and lack of normalization of precursor quantification in the consensus workflow.

#### ‘*Candidatus* Scalindua brodae’ proteome

Cells were pelleted and lysed under native conditions with hypotonic buffer (10 mM HEPES, 10 mM NaCl, 1.5 mM MgCl_2_, 2 mM EDTA, 0.1% NP-40, Roche Mini protease inhibitor) and a probe sonicator (20% power, 10 s with 1 s pulse, 5 rounds) on ice. Lysates were upconcentrated and buffer exchanged with spin filters (Amicon, 3 kDa cut-off, UFC500324, Merck Millipore) to 50 mM HEPES pH 7.8, and their concentration was determined by Nanodrop. From then on, the standard proteomics sample preparation was followed, starting with 50 μg of proteome. Proteins were reduced, alkylated and digested as described above. Assuming no losses, 1 μg of peptides was acidified and loaded on EvoTips with the low-input protocol. The samples were analysed with EvoSep One liquid chromatography platform, in line with an Orbitrap Eclipse mass spectrometer equipped with a FAIMSpro device. Peptides were separated with a PepSep C18 15 cm × 150 μm, 1.9 μm (PepSep, 1893471), over 44 min with the standard 30SPD method. Peptides were ionized with nanospray ionization with an 10 μm emitter (PepSep, 1893527), and spray voltage of 2,300 V in positive-ion mode, and ion transfer tube of 240 °C. Spectra were acquired in data-dependent acquisition mode, under 2 different compensation voltages of −50 and −70 V, with identical settings. The cycle time was set to 1.2 s, with MS1 spectra acquired with 60,000 resolution, and a maximum injection time of 118 s. MS2 spectra were acquired with an isolation window of 1.6 *m*/*z*, normalized HCD of 30%, with otherwise similar settings as above. Data analysis was performed as above, with the only differences being the use of the putative proteome ‘*Candidatus* Scalindua brodae’ database, assembled from metagenomics data (Uniprot Trembl, UP000030652, 4,014 sequences, accessed 28 February 2023), and lack of normalization of precursor quantification in the consensus workflow. In a secondary search, the raw data were searched against the ‘*Candidatus* Scalindua brodae’ proteome as above, along with the proteomes of *Candidatus* Kuenenia stuttgartiensis (UP000221734, 3,801 sequences, accessed 27 July 2023), *Candidatus* Scalindua rubra (UP000094056, 5,207 sequences, accessed 27 July 2023) and the *Candidatus* Scalindua profunda metagenome from a previous study (23,834 sequences)^76^.

#### GluC degradome and PRM monitoring

HeLa cell lysates were extracted as in the HeLa proteome section. Six aliquots of 20 μg of lysate were resuspended in 100 mM HEPES, pH 7.8 to reduce the GuHCl concentration to 0.5 M. Two-hundred nanograms of GluC endopeptidase (V1651, Promega) was added to 3 out of the 6 samples to a protease to proteome ratio of 1:100 ratio, and all samples were incubated at 37 °C, 450 rpm, for 20 min. Samples were reduced, alkylated and digested with trypsin as described previously. The next day, volume equivalent to 1 μg from each sample, assuming no losses, was loaded on EvoTips as described above, and samples were analysed using the EvoSep One liquid chromatography platform, in line with an Orbitrap Eclipse mass spectrometer equipped with a FAIMSpro device. Peptides were eluted from a PepSep C18 column (15 cm × 75 μm, 1.9 μm PepSep, 1893473) over 58 min with the Whisper100 20SPD method. Scans were acquired with the same settings as in the HeLa proteome single-shot analysis. Data analysis was performed as above, with use of the human database for the HeLa proteome searches, semi-tryptic search and precursor quantification normalized on the total peptide amount from each sample in the consensus workflow.

PRM assays were designed for representative peptides detected by IN with high confidence, but not with the database search. Peptide sequences were imported in Skyline^77^, and an inclusion list with the precursor masses was exported. The inclusion list was used to create a PRM monitoring method with a targeted mass inclusion filter for acquisition of MS/MS scans. GluC degradome samples were analysed with the same set-up as in shotgun proteomics and the same FAIMS compensation voltages. Scans were acquired with 60,000 resolution for MS1 and 15,000 resolution for MS2, and a cycle time of 1 s for each FAIMS compensation voltage, with otherwise similar settings with the shotgun proteomics experiment. Results were analysed and visualized with Skyline.

#### Wound exudate pathogen validation

The wound exudates were extracted from patient wound dressings as described in ref. 50. PCR amplification of the 16S rRNA gene was performed using MyTaq Red Mix (Bioline) in a final reaction volume of 20 μl, with 2 sets of primers: 1 specific for the 16S rRNA gene of *E. coli* (expected amplicon size 544 bp; annealing temperature 60 °C)^78^ and another specific for the 16S rRNA gene of *Pseudomonas* spp. (expected amplicon size 544 bp; *T*_m_ 54 °C)^79^. Each reaction contained 10 μl of MyTaq Red Mix, 1 μl of each primer, 2 μl of the sample, and nuclease-free water to adjust the final volume. As positive controls, 1 μl of a colony dilution prepared from fresh colonies of *E. coli* BL21(DE3) or *P. aeruginosa* PA01 was used. PCR was conducted with an initial denaturation at 95 °C for 3 min, followed by 35 cycles of 95 °C for 20 s, annealing at the primer-specific *T*_m_ (60 °C or 54 °C) for 20 s (Supplementary Table 3), and extension at 72 °C for 20 s, with a final extension at 72 °C for 90 s. Post-PCR, 6 μl of each reaction product was loaded onto a 1% (w/v) agarose gel prepared in 1X TAE buffer containing SYBR Safe (S33102, ThermoFisher). Electrophoresis was carried out at 100 V for 45 min, and DNA bands were visualized under ultraviolet light using a gel documentation system, with a 1 kb Plus DNA ladder (ThermoFisher) as the molecular weight reference.

#### External dataset analysis

The raw data from a snake venom proteomics dataset were downloaded and reanalysed using the Uniprot database sequences for the serpentes order (331,759 sequences, accessed 5 September 2022), similar to the original study. Data were analysed with Proteome Discoverer v2.4 and the Sequest HT search engine, with all files included in the same analysis, normalization on total peptide amount and precursor quantification, with other settings similar to other datasets. The herceptin dataset was downloaded and analysed similarly. However, the raw data from the six different proteases were searched separately, and no precursor or normalization was performed. The same fasta database as in the original study was used for PSM detection. Search results were then combined for prediction and evaluation.

The immunopeptidomics dataset was reprocessed with the same proteome database as in the original paper with MSFragger^13^ and the FragPipe v21.1 pipeline with the non-specific HLA workflow, and otherwise default settings. MSBooster^80^ was used for rescoring with deep learning prediction, and Percolator was used for PSM FDR control, while no FDR control was used on the protein level.

The wound fluid dataset was downloaded and searched with the same human database as used for the HeLa proteome and GluC degradomics experiments. Both raw data files were analysed in the same search in Proteome Discoverer v2.4, with total peptide amount normalization and precursor quantification. In the secondary search results, the same human proteome as well as protein sequences downloaded from the Uniprot database for the pathogens of interest *Citrobacter* sp. (UP000682339, 3,414 sequences), *P. aeruginosa* (UP000002438, 5,564 sequences), *S. aureus* (UP000008816, 2,889 sequences) and *E. coli* (UP000000625, 4,403 sequences) were used for PSM detection.

### Reporting summary

Further information on research design is available in the Nature Portfolio Reporting Summary linked to this article.

## Extended Data

**Extended Data Fig. 2:**
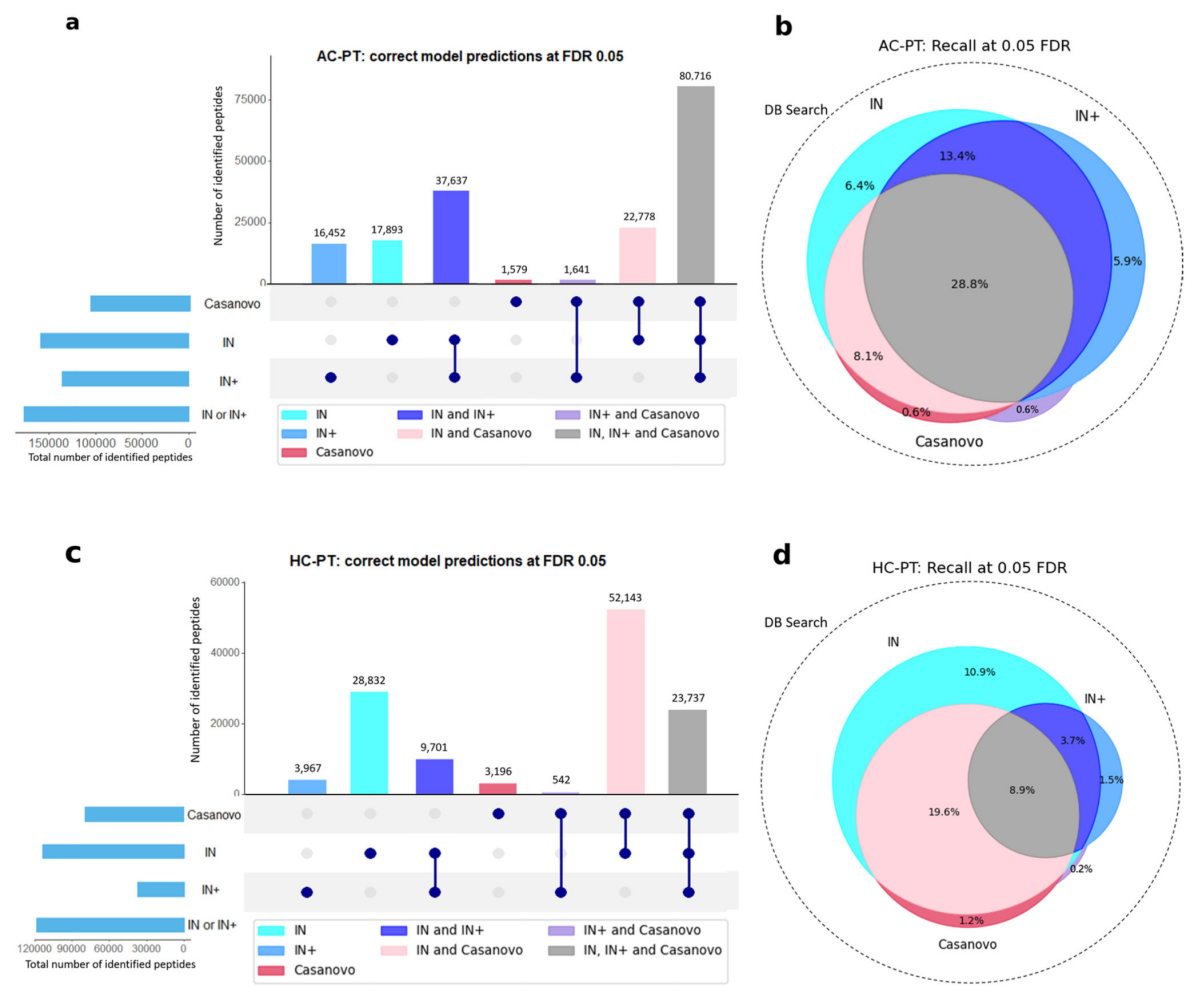
Overlaps between IN, IN+ and Casanovo’s correct predictions at 0.05 FDR for AC-PT and HC-PT. **a**, Peptide-level UpSet plot illustrating the intersection of correct predictions made by the IN, IN+, and Casanovo models on the AC-PT dataset, when evaluated at a false discovery rate (FDR) of 0.05. **b**, Peptide-level Venn Diagram illustrating the same intersections as figure a, but displaying them as percentages (recall) of the DB search ground truth dataset, which is illustrated by the area of the circle with the dotted edge. Areas in the Venn diagram are approximate, due to the imperfection of the Venn algorithm. **c**, Equivalent of figure a, for the HC-PT dataset **d**, Equivalent of figure b, for the HC-PT dataset.

**Extended Data Fig. 3:**
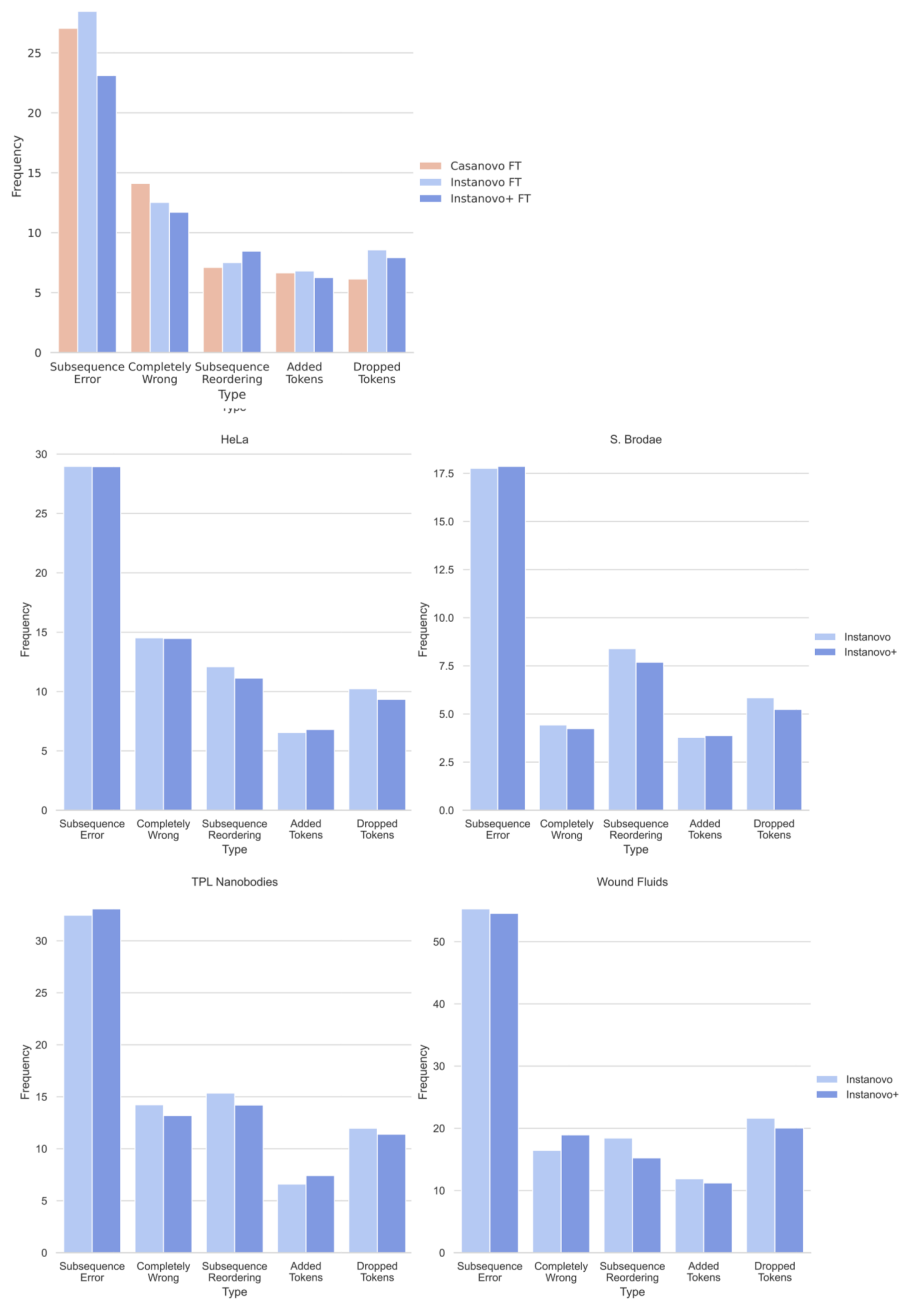
Error analysis for a selection of evaluation datasets. Top left: Comparison of Casanovo, IN, and IN+ predictions errors in the nine-species dataset. Most errors are caused by a few errors in the overall amino acid sequence for all models. Bottom: Comparison of IN and IN+ errors in 4 out of the 8 biological datasets.

**Extended Data Fig. 4:**
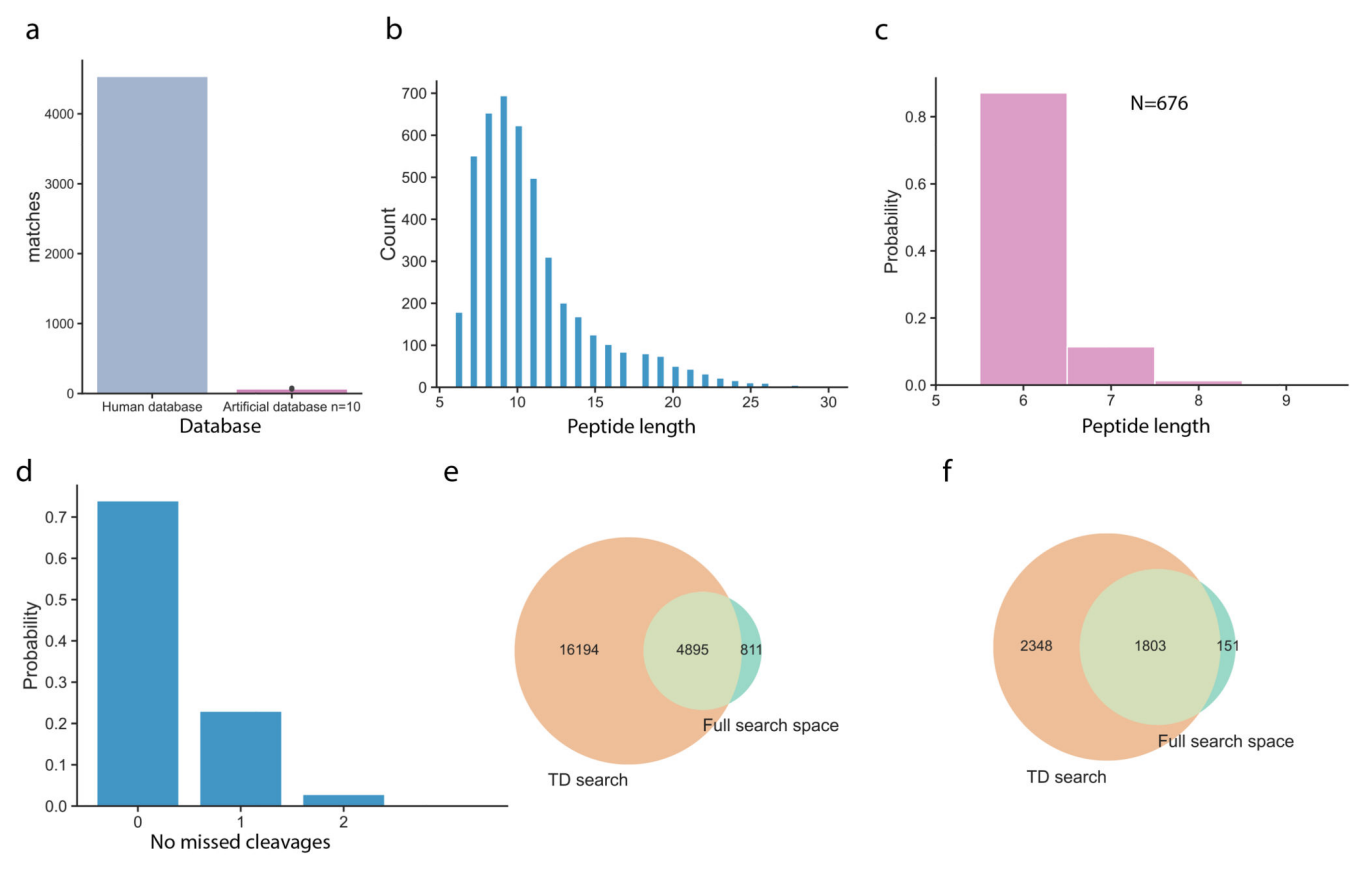
Extended figure for HeLa proteome analysis. **a**, Human database vs artificially generated database peptide matches comparison, database search space. **b**, Peptide length distribution in human proteome mapped predictions. **c**, Length of prediction matches in 10 artificially and randomly generated databases. **d**, Distribution of missed cleavages in full space predictions at 5% FDR. **e**, Venn diagram of peptide sequences mapping to the human proteome, identified with database search and sequences predicted by Instanovo in the full search space. **f**, Proteins identified from peptide sequences of database search PSMs or InstaNovo predictions in the full search space at 5% FDR.

**Extended Data Fig. 5:**
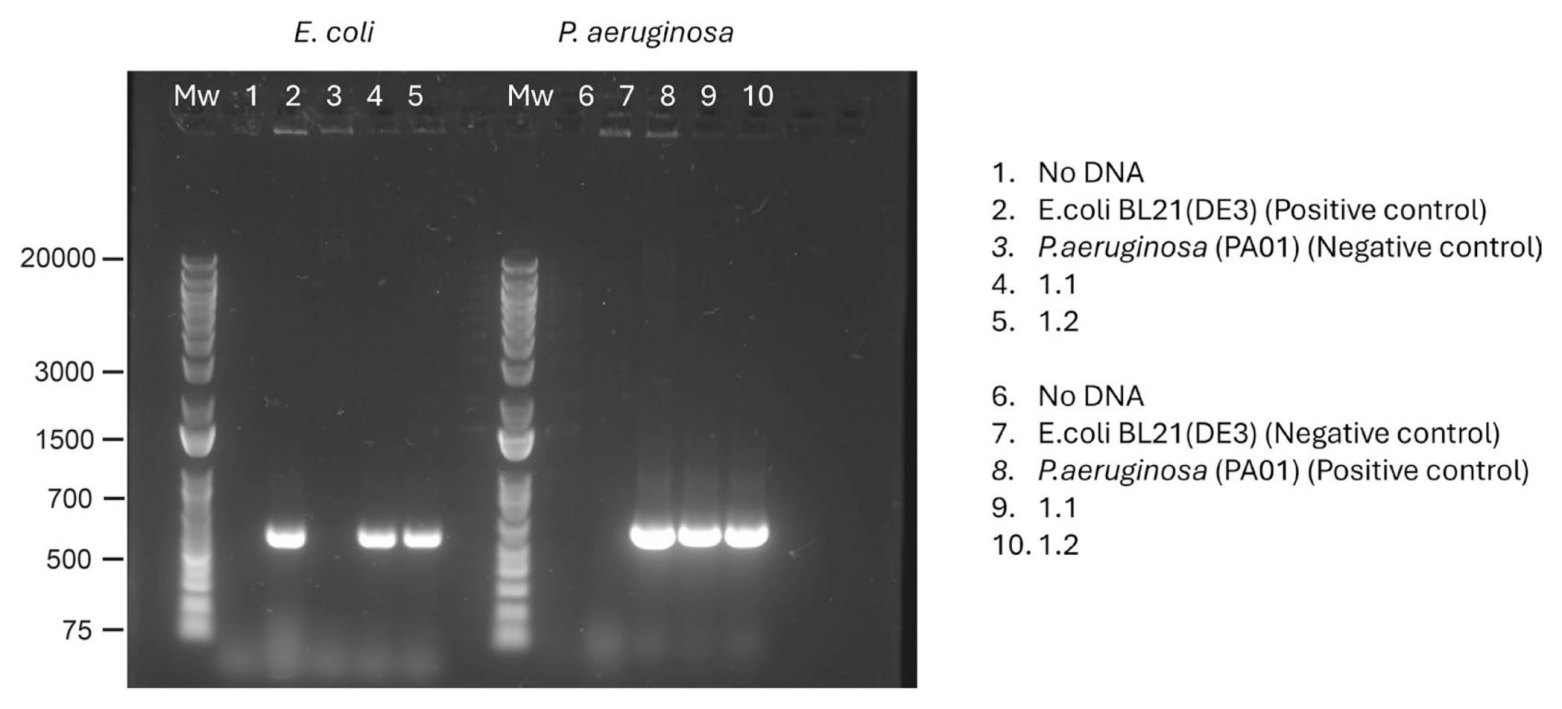
16s rRNA PCR of human pathogens in wound fluids. *Escherichia coli* and *Pseudomonas aeruginosa* primers were designed for the 16s rRNA genes, and a PCR amplification assay was performed to detect these organisms in the patient wound fluids, as a validation of our de novo peptide sequencing results.

**Extended Data Fig. 6:**
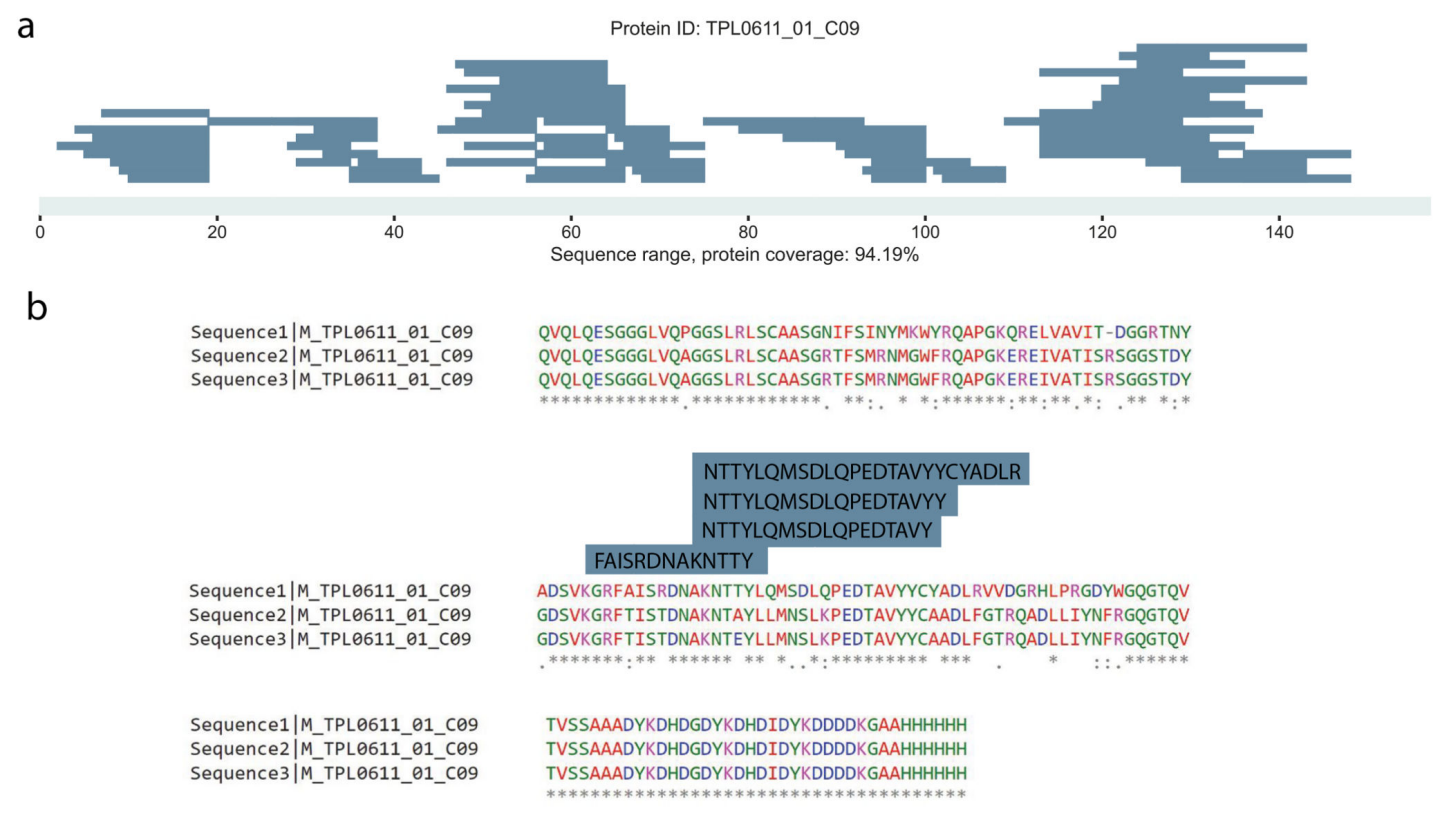
Direct sequencing and conflict resolution with InstaNovo. **a**, Nanobody TPL0611 01 C09 coverage and sequencing depth with unique peptides predicted at 5% FDR. **b**, Alignment of three separate sequencing runs on cells expressing the C09 nanobody, annotated with unique peptide sequences predicted with InstaNovo, mapping to one of the areas where there was ambiguity in determination of the sequence with genome sequencing methods.

**Extended Data Fig. 7:**
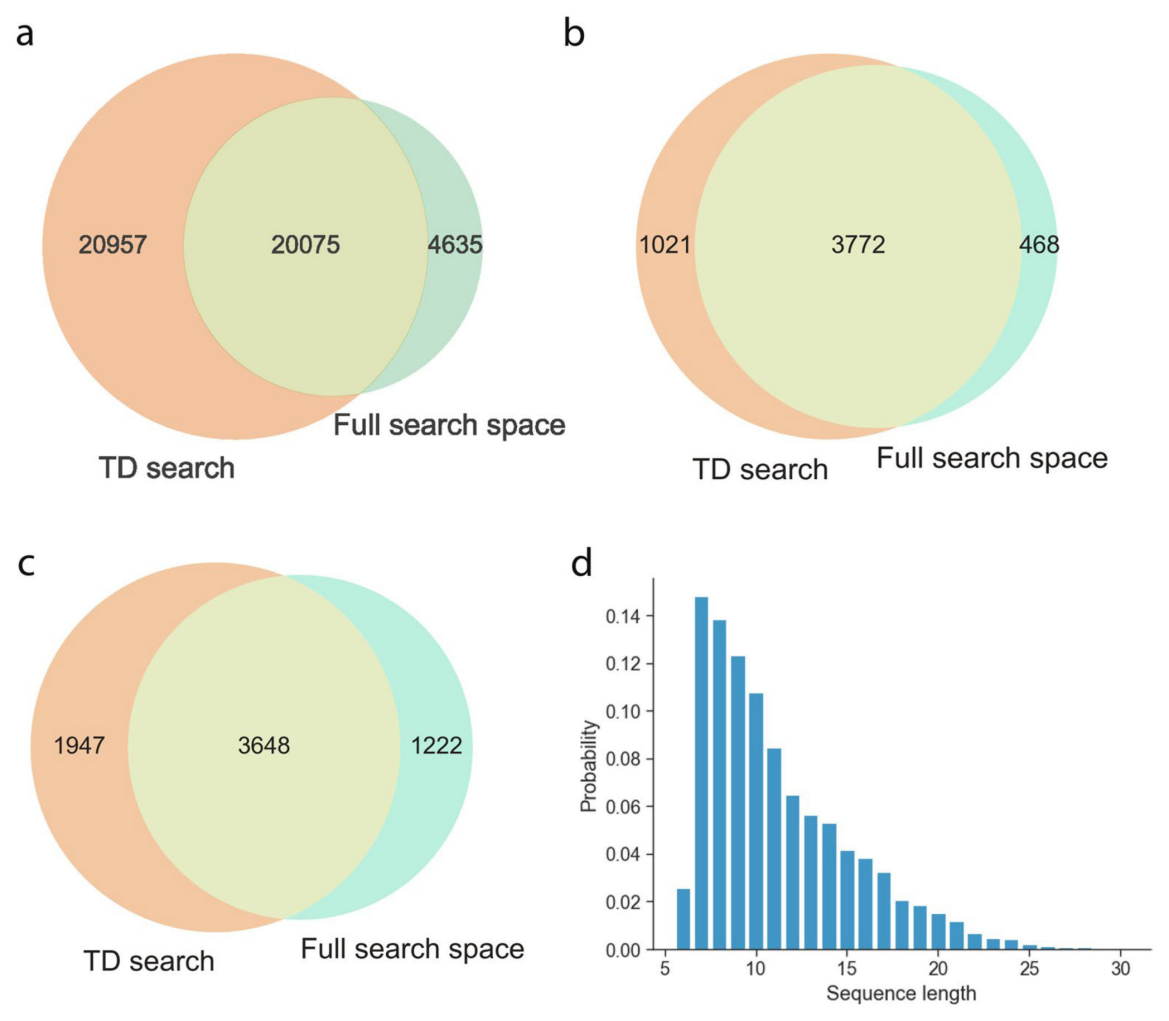
InstaNovo accurately predicts and expands detection rates in HeLa GluC degradome. **a**, Unique peptide sequences of database search and InstaNovo predicted peptides matching to the human reference proteome at 5% FDR. **b**, Proteins detected by predicted peptide sequences InstaNovo at 5% FDR. **c**, GluC candidate cleavages identified at 5% FDR (preceded by glutamate residue). **d**, Sequence length distribution for GluC generated peptides (preceded by glutamate residue).

**Extended Data Table 1:** Database search results

*Dataset*	*MS/MS*	*TD-search PSMs*	*TD-search Peptides*	*TD-search Proteins*
HeLa single shot	463,777	25,107	21,104	3,783
Nanobodies	257,701	23,147	5,897	924
Herceptin	58,609	1,796	129	2
Wound fluids	100,054	20,699	8,307	1,096
*Candidatus* “Scalindua brodae”	26,099	9,068	7,881	1,694
Snake venoms	558,247	21,257	3,446	610
Immunopeptidome	404,062	99,178	20,904	5,948
HeLa degradomics	204,831	115,470	41,483	4,438

Database search results for the datasets used in this study at 1% FDR, except for immunopeptidomics (no protein FDR).

**Extended Data Table 2:** InstaNovo evaluation results on all datasets

*Dataset*	A A-level performance			Peptide-level performance	
	*Error rate*	*Precision*	*Accuracy*	*Accuracy*	*AUG*
HeLa single-shot	0.330 ± 0.005	0.609 ± 0.007	0.608 ± 0.007	0.503 ± 0.007	0.465 ± 0.008
Immunopeptidomics	0.211 ± 0.012	0.778 ± 0.020	0.779 ± 0.020	0.581 ± 0.036	0.532 ± 0.045
*Candidatus* “Scalindua brodae”	0.204 ± 0.004	0.815 ± 0.008	0.815 ± 0.008	0.724 ± 0.010	0.697 ± 0.011
Snake Venoms	0.494 ± 0.006	0.396 ± 0.008	0.398 ± 0.008	0.196 ± 0.008	0.167 ± 0.009
Nanobodies	0.339 ± 0.004	0.597 ± 0.006	0.595 ± 0.006	0.447 ± 0.007	0.412 ± 0.007
Wound Fluids	0.467 ± 0.009	0.411 ± 0.012	0.406 ± 0.012	0.225 ± 0.014	0.190 ± 0.014
HeLa degradome	0.178 ± 0.001	0.798 ± 0.002	0.798 ± 0.002	0.695 ± 0.003	0.676 ± 0.003
Herceptin	0.215 ± 0.015	0.659 ± 0.029	0.658 ± 0.029	0.494 ± 0.035	0.472 ± 0.037
Yeast	0.279	0.709	0.626	0.559	0.528
Bacillus	0.197	0.762	0.721	0.624	0.595
Mouse	0.224	0.695	0.692	0.466	0.428
HC-PT*	0.279	0.687	0.685	0.573	0.550
AC-PT*	0.193	0.794	0.794	0.685	0.666
*mean*	0.278	0.670	0.660	0.521	0.491
*std*	0.104	0.138	0.136	0.163	0.166

Confidence intervals are calculated as ± 1.96 × $\hat{se_{B}}$ where $\hat{se_{B}}$ is a bootstrap standard error estimated from 10,000 replicates. *We do not calculate bootstrap standard errors for the ProteomeTools datasets because their size makes it prohibitively costly but also implies the standard errors would be very small. We exclude the bootstrap standard errors for the nine-species dataset on the same basis.

**Extended Data Table 3:** InstaNovo+ evaluation results on all datasets

*Dataset*	A A-level performance			Peptide-level performance	
	*Error rate*	*Precision*	*Accuracy*	*Accuracy*	*AUG*
HeLa single-shot	0.321 ± 0.004	0.617 ± 0.007	0.616 ± 0.007	0.517 ± 0.007	0.477 ± 0.008
Immunopeptidomics	0.161 ± 0.012	0.839 ± 0.022	0.839 ± 0.022	0.697 ± 0.036	0.644 ± 0.044
*Candidatus* “Scalindua brodae”	0.187 ± 0.004	0.821 ± 0.008	0.820 ± 0.008	0.736 ± 0.009	0.697 ± 0.011
Snake Venoms	0.493 ± 0.006	0.393 ± 0.008	0.395 ± 0.008	0.198 ± 0.008	0.137 ± 0.009
Nanobodies	0.329 ± 0.004	0.608 ± 0.006	0.606 ± 0.006	0.464 ± 0.007	0.417 ± 0.008
Wound Fluids	0.467 ± 0.009	0.412 ± 0.012	0.406 ± 0.012	0.229 ± 0.013	0.166 ± 0.014
HeLa degradome	0.163 ± 0.001	0.811 ± 0.002	0.810 ± 0.002	0.719 ± 0.003	0.689 ± 0.003
Herceptin	0.192 ± 0.014	0.710 ± 0.028	0.709 ± 0.028	0.562 ± 0.034	0.526 ± 0.038
Yeast	0.256	0.755	0.667	0.624	0.598
Bacillus	0.180	0.796	0.753	0.674	0.650
Mouse	0.209	0.726	0.722	0.490	0.431
HC-PT*	0.268	0.696	0.694	0.589	0.542
AC-PT*	0.178	0.809	0.809	0.710	0.680
*mean*	0.262	0.692	0.680	0.555	0.512
*std*	0.112	0.148	0.145	0.176	0.186

Confidence intervals are calculated as ± 1.96 × $\hat{se_{B}}$ where $\hat{se_{B}}$ is a bootstrap standard error estimated from 10,000 replicates. *We do not calculate bootstrap standard errors for the ProteomeTools datasets because their size makes it prohibitively costly but also implies the standard errors would be very small. We exclude the bootstrap standard errors for the nine-species dataset on the same basis.

## Supplementary Material

**Supplementary information** The online version contains supplementary material available at https://doi.org/10.1038/s42256-025-01019-5.

Reporting Summary

Supplementary Material

## Data Availability

Raw data and search results used for evaluation, and public datasets used or datasets generated in this study, have been deposited to the ProteomeXchange Consortium via the PRIDE^81^ partner repository with the dataset identifier PXD044934. Additional files relating to pre-processed results used for training and metric evaluation have also been uploaded in the same archive repository. Supplementary files supporting the data preprocessing, tool usage and analysis performed on eight different application-centric datasets have been deposited on figshare at https://doi.org/10.6084/m9.figshare.24173889 (ref. 82). The ProteomeTools datasets used to train the models in this study can be found in the PRIDE repository with identifiers PXD004732 (Part I), PXD010595 (Part II) and PXD021013 (Part III). The nine-species dataset^30^ is available through the MassIVE repository with dataset identifier MSV000081382. The immunopeptidomics dataset^83^ used for model evaluation can be found in the PRIDE repository with identifier PXD006939. Snake venom files and search results^51^ can be found in the PRIDE repository with identifier PXD036161. The wound exudate files and search results^50^ are available in PanoramaWeb with dataset identifier PXD025748. The herceptin dataset^49^ is available on figshare at https://doi.org/10.6084/m9.figshare.21394143 (ref. 84).

## Code availability

InstaNovo and InstaNovo+ are available at https://github.com/instadeepai/InstaNovo and on Zenodo at https://doi.org/10.5281/zenodo.14712453 (ref. 85) along with model checkpoints and a Google Colab notebook for easy experimentation, demonstration and integration into research workflows. In addition, a user-friendly website is linked from the GitHub repository where users can upload their data and receive predictions directly, making the models accessible without requiring local set-up. Furthermore, we have made the nine-species dataset^30^ also available at https://huggingface.co/datasets/InstaDee-pAI/ms_ninespecies_benchmark (https://doi.org/10.57967/hf/3821)^86^, and the high-confidence ProteomeTools^36^ dataset available at https://huggingface.co/datasets/InstaDeepAI/ms_proteometools (https://doi.org/10.57967/hf/3822)^87^. Custom scripts used for data analysis and visualization are available on figshare (https://doi.org/10.6084/m9.figshare.24173889.v1) (ref. 82).
