# Supplementary material for: InstaNovo enables diffusion-powered de novo peptide sequencing in large-scale proteomics experiments
Source: Nat Mach Intell. Author manuscript; Available in PMC 2026 Mar 16. (PMC7618892; doi:10.1038/s42256-025-01019-5)
Supplement: Reporting Summary [file EMS212824-supplement-Reporting_Summary.pdf]

nature portfolio

Corresponding author(s): TPJ, KK, KE

Last updated by author(s): Jan 21, 2025

Reporting Summary

Nature Portfolio wishes to improve the reproducibility of the work that we publish. This form provides structure for consistency and transparency in reporting. For further information on Nature Portfolio policies, see our Editorial Policies and the Editorial Policy Checklist.

Statistics

For all statistical analyses, confirm that the following items are present in the figure legend, table legend, main text, or Methods section.

n/a	Confirmed	
☐	☒	The exact sample size (*n*) for each experimental group/condition, given as a discrete number and unit of measurement
☐	☒	A statement on whether measurements were taken from distinct samples or whether the same sample was measured repeatedly
☐	☒	The statistical test(s) used AND whether they are one- or two-sided<br>*Only common tests should be described solely by name; describe more complex techniques in the Methods section.*
☒	☐	A description of all covariates tested
☒	☐	A description of any assumptions or corrections, such as tests of normality and adjustment for multiple comparisons
☒	☐	A full description of the statistical parameters including central tendency (e.g. means) or other basic estimates (e.g. regression coefficient) AND variation (e.g. standard deviation) or associated estimates of uncertainty (e.g. confidence intervals)
☒	☐	For null hypothesis testing, the test statistic (e.g. *F*, *t*, *r*) with confidence intervals, effect sizes, degrees of freedom and *P* value noted<br>*Give P values as exact values whenever suitable.*
☒	☐	For Bayesian analysis, information on the choice of priors and Markov chain Monte Carlo settings
☒	☐	For hierarchical and complex designs, identification of the appropriate level for tests and full reporting of outcomes
☒	☐	Estimates of effect sizes (e.g. Cohen's *d*, Pearson's *r*), indicating how they were calculated

Our web collection on statistics for biologists contains articles on many of the points above.

Software and code

Policy information about availability of computer code

Data collection	All commercial, open source and custom code are provided in the manuscript and the related code repository.
Data analysis	Guidelines for data collection, preprocessing and executing the code, along with custom scripts used in the analysis are available in the code repository or upon request.

For manuscripts utilizing custom algorithms or software that are central to the research but not yet described in published literature, software must be made available to editors and reviewers. We strongly encourage code deposition in a community repository (e.g. GitHub). See the Nature Portfolio guidelines for submitting code & software for further information.

Data

Policy information about availability of data

All manuscripts must include a data availability statement. This statement should provide the following information, where applicable:

- Accession codes, unique identifiers, or web links for publicly available datasets
- A description of any restrictions on data availability
- For clinical datasets or third party data, please ensure that the statement adheres to our policy

Data used or generated in this study are available in the core repository or upon request.

Research involving human participants, their data, or biological material

Policy information about studies with human participants or human data. See also policy information about sex, gender (identity/presentation), and sexual orientation and race, ethnicity and racism.

Reporting on sex and gender	Not applicable.
Reporting on race, ethnicity, or other socially relevant groupings	Not applicable.
Population characteristics	Not applicable.
Recruitment	Not applicable.
Ethics oversight	The study was carried out under the FAIR principle guidelines, as well as standard ethical guidelines from the Technical University of Denmark

Note that full information on the approval of the study protocol must also be provided in the manuscript.

Field-specific reporting

Please select the one below that is the best fit for your research. If you are not sure, read the appropriate sections before making your selection.

☒ Life sciences ☐ Behavioural & social sciences ☐ Ecological, evolutionary & environmental sciences

For a reference copy of the document with all sections, see nature.com/documents/nr-reporting-summary-flat.pdf

Life sciences study design

All studies must disclose on these points even when the disclosure is negative.

Sample size	Statistics were performed in two cases. The first was the comparison of peptide spectrum matches of a HeLa proteome analyzed in a single shot mass spectrometry analysis, to the human protein database (Uniprot, canonical Homo Sapiens proteins) or to randomly generated, equivalent in number of entries and protein size artificial databases. The sample size of N=10 was used to generate 10 artificial databases, and the population of matches was compared to the mean (human database matches) with an one-sample T-test. No power analysis was performed, but it was deemed sufficient given the statistic score and probability value.<br>The second was the determination of GluC cleavages from the HeLa degradome samples, where N=3 sample size was used for a shotgun analysis of GluC treated HeLa proteomes, and control (non-treated) HeLa proteomes. Only peptide predictions with identifications both from the model and the associated database search were used, with quantification values present in all replicates. Replicates used were biological and not technical. A two sample independent T-test analysis was performed on the log2 transformed peptide quantification values transferred from the database search software, for the two conditions. The peptides were ranked based on statistical significance, and a panel of the top hits was selected and monitored with targeted proteomics. No power analysis was performed prior to the statistical test, and no statistics were performed in the targeted proteomics analysis.
Data exclusions	Only peptide predictions with identifications both from the model and the associated database search were used, with quantification values present in all replicates.
Replication	Three biological replicates were used in the analysis of HeLa degradomes. All other analyses was performed in single shot analytical runs of proteomes from various sources.
Randomization	Not applicable.
Blinding	Not applicable.

Reporting for specific materials, systems and methods

We require information from authors about some types of materials, experimental systems and methods used in many studies. Here, indicate whether each material, system or method listed is relevant to your study. If you are not sure if a list item applies to your research, read the appropriate section before selecting a response.

Materials & experimental systems

n/a	Involved in the study	
☐	☒	Antibodies
☐	☒	Eukaryotic cell lines
☒	☐	Palaeontology and archaeology
☐	☒	Animals and other organisms
☒	☐	Clinical data
☒	☐	Dual use research of concern
☒	☐	Plants

Methods

n/a	Involved in the study	
☒	☐	ChIP-seq
☒	☐	Flow cytometry
☒	☐	MRI-based neuroimaging

Antibodies

Antibodies used	No reagent antibodies were used in this study. The nanobodies included in this study were discovered using phage display technology. Briefly, camelids were immunised with whole venoms from either 8 viperid snake species or 18 elapid snake species, followed by the construction of immune nanobody displaying phage libraries (VIB Nanobody Core, Brussels). A detailed description of how they were discovered can be found in the methods section.
Validation	N/A

Eukaryotic cell lines

Policy information about cell lines and Sex and Gender in Research

Cell line source(s)	The HeLa cell line was purchased from Thermo Fisher Scientific (lot. no. in manuscript). The S. brodae pelleted co-culture was obtained from S.v.B. and S.J.J.B, who acquired it from collaborators as mentioned in the acknowledgements.
Authentication	HeLa cell lines were tested for mycoplasma and have been determined negative. No such test was performed in the S. brodae co-culture.
Mycoplasma contamination	HeLa cell lines were tested for mycoplasma and have been determined negative. No such test was performed in the S. brodae co-culture.
Commonly misidentified lines (See ICLAC register)	Not applicable.

Animals and other research organisms

Policy information about studies involving animals; ARRIVE guidelines recommended for reporting animal research, and Sex and Gender in Research

Laboratory animals	No laboratory animals were used in this study.
Wild animals	Not applicable.
Reporting on sex	Not applicable.
Field-collected samples	Not applicable.
Ethics oversight	XXXXXXXXXXXX

Note that full information on the approval of the study protocol must also be provided in the manuscript.
