# Supplementary material for: InstaNovo enables diffusion-powered de novo peptide sequencing in large-scale proteomics experiments
Source: Nat Mach Intell. Author manuscript; Available in PMC 2026 Mar 16. (PMC7618892; doi:10.1038/s42256-025-01019-5)
Supplement: Supplementary Material [file EMS212824-supplement-Supplementary_Material.pdf]

nature machine intelligence

Supplementary information

https://doi.org/10.1038/s42256-025-01019-5

InstaNovo enables diffusion-powered de novo peptide sequencing in large-scale proteomics experiments

In the format provided by the authors and unedited

Supplementary Figures

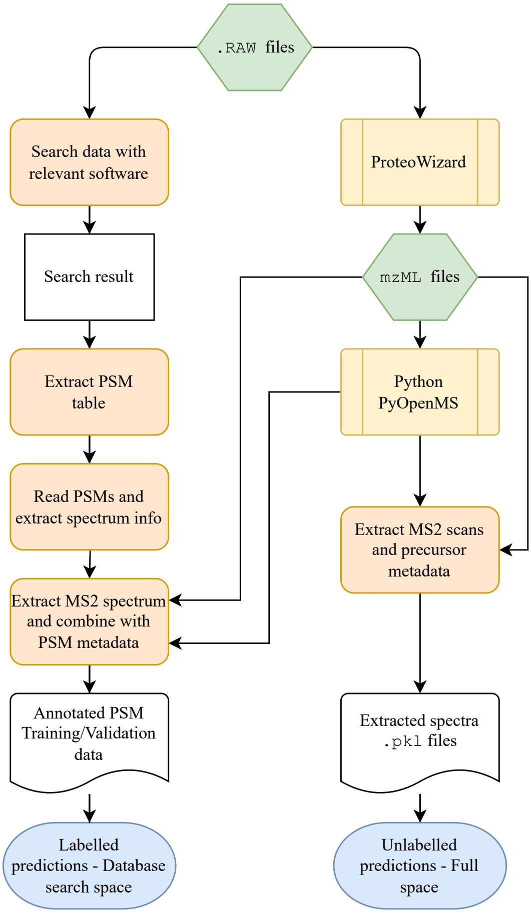

Supplementary Fig. 1 Detailed workflow for dataset extractions and preprocessing. Thermo `.raw files` were searched against a proteome database with Proteome Discoverer or MaxQuant. MSConvert from the Proteowizard tool suite was used to convert `.raw` files to mzML. The mzML files were used to extract m/z and intensity vectors as well as associated metadata with pyOpenMS. Two datasets were created, the database search space containing only scans that were matched to peptide hits (PSMs) from the database searches, and the full search space which contained all MS/MS scans from all `.raw` files of each experiment.

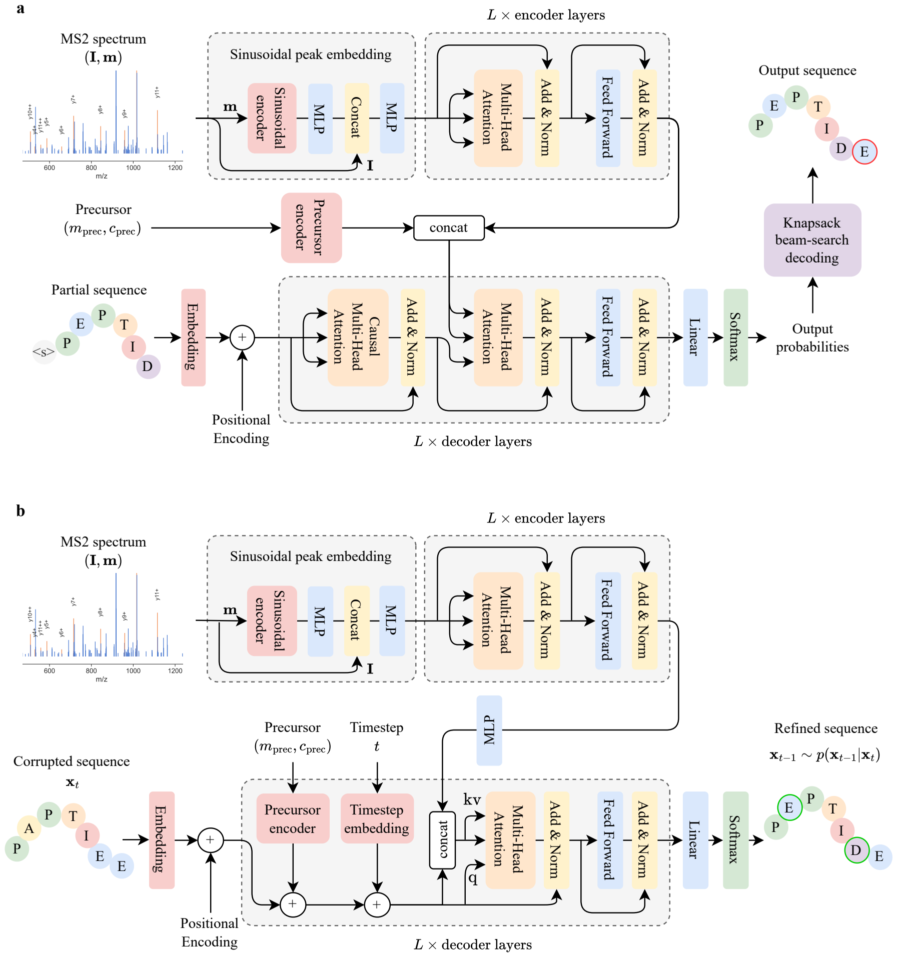

Supplementary Fig. 2 Detailed model architecture and description of InstaNovo and InstaNovo+ model. a, InstaNovo model architecture. The precursor information may also be provided as a start-of-sequence embedding at the input to the decoder layers. **b,** InstaNovo+ model architecture.

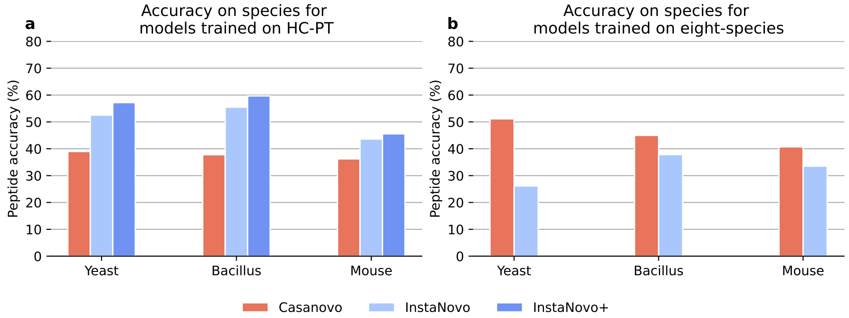

Supplementary Fig. 3 Peptide accuracy on yeast, bacillus and mouse for models trained on the HC-PT dataset, and nine-species dataset, respectively. a, Comparison of peptide accuracy for Casanovo, IN, and IN+ trained on the HC-PT dataset. We see increasing accuracy when utilising IN and IN+. **b,** Comparison of peptide accuracy for Casanovo, and IN trained on the nine-species dataset, excluding the respective evaluation datasets. Note that IN and IN+ were not designed to be trained on small datasets, such as the nine-species dataset, and are expected to perform poorly. Hence, we exclude IN+ from this evaluation, and include IN as an illustration.

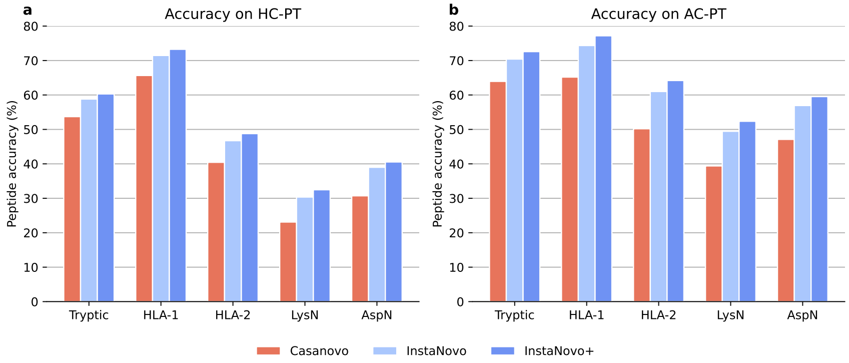

Supplementary Fig. 4 Peptide accuracy on HC-PT and AC-PT grouped by the type of peptide.

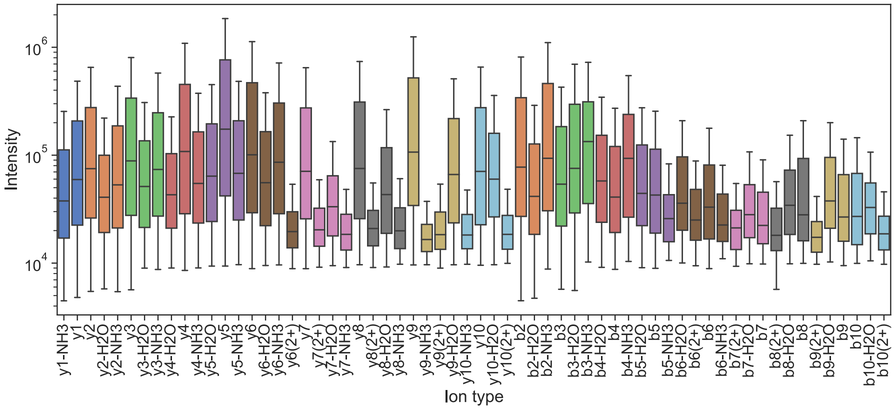

Supplementary Fig. 5 Fragment ion log intensity from a selected analytical run from the training data, showing all ion types from identified PSMs (n=32041, median as center line, 25th to 75th percentiles as bounds of the box, whiskers extending to 1.5 times the interquartile range from the bounds of the box, with minima and maxima beyond the whiskers plotted as individual points).

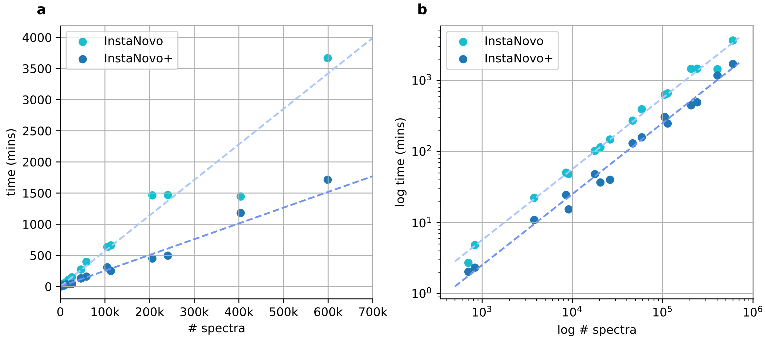

Supplementary Fig. 6 Runtime analysis of InstaNovo and InstaNovo+ showing a linear relationship. a, Inference time in minutes compared to the number of spectra. **b,** Comparison of runtime in the log-domain. The dashed lines in **a** and **b** depict the mean runtime per spectra. Presented runtimes were performed on hardware setup B (Supplementary Table 1). Hardware setup A saw a 2× average runtime improvement over setup B in knapsack decoding, due to poor performance of the server-grade CPU becoming a bottleneck in beam-search knapsack. When using InstaNovo predictions as a starting point for InstaNovo+, the total runtime would be the sum of the two models.

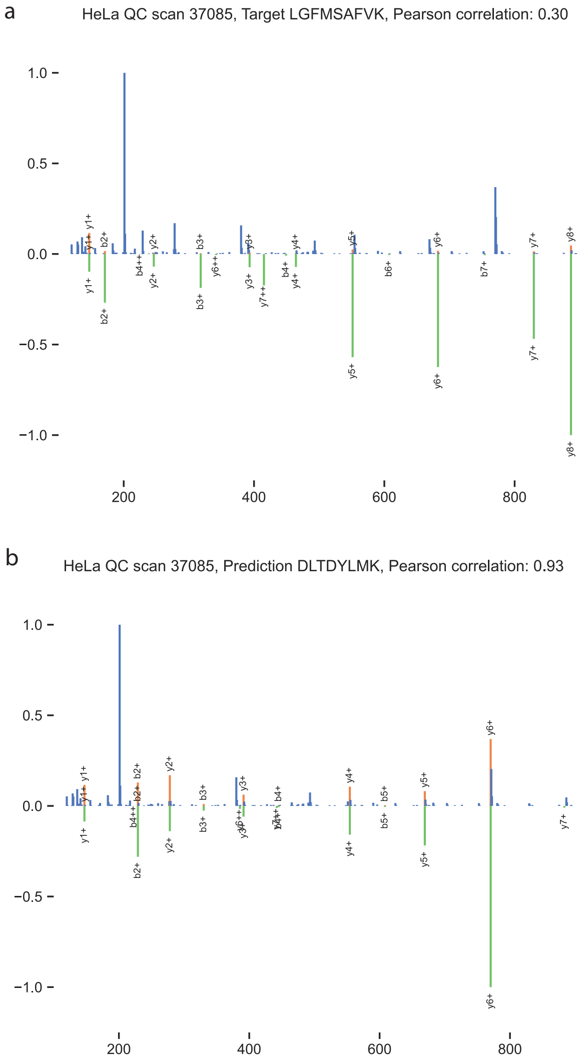

Supplementary Fig. 7 InstaNovo predicted sequence correlates better with observed spectrum than with database search PSM. a, Mirror plot for database search PSM sequence LGFMSAFVK in HeLa QC dataset, scan number 37,085. Top, experimental spectrum, bottom, Prosit predicted spectrum for the same sequence. **b,** Similar plot for predicted sequence, exhibiting higher similarity with experimental spectrum.

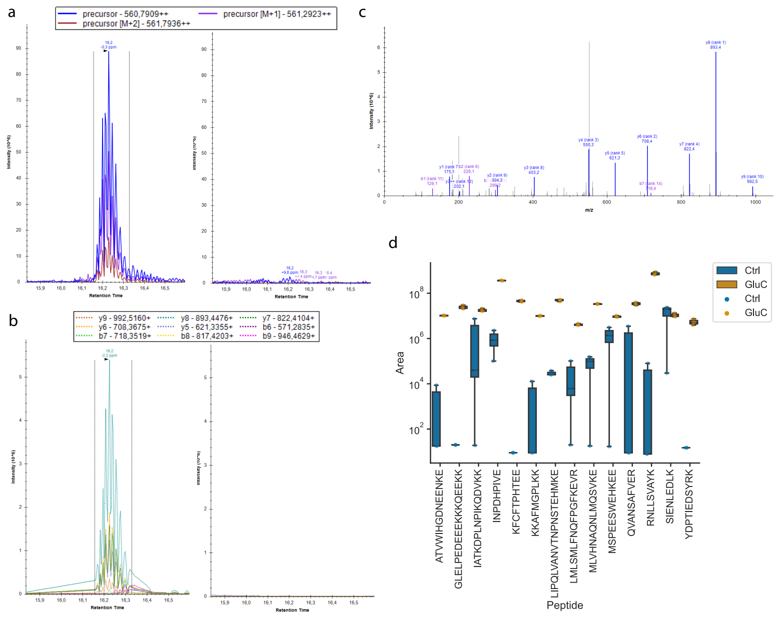

Supplementary Fig. 8 Targeted proteomics in GluC dataset. a, Monitoring of precursor mass for peptide QVANSAFVER in one GluC digested (left) and control (right) replicates. **b,** Monitoring of peptide transitions with fragment ion masses for the same peptide and replicates. **c,** Experimental spectrum at the apex of the transition peaks in the GluC digested replicate for the same peptide. **d,** Boxplot and striplot visualisation of the sum of fragment ion areas for selected peptides monitored with targeted proteomics (n=3, median as center line, 25th to 75th percentiles as bounds of the box, whiskers extending to 1.5 times the interquartile range from the bounds of the box, with minima and maxima beyond the whiskers plotted as individual points).

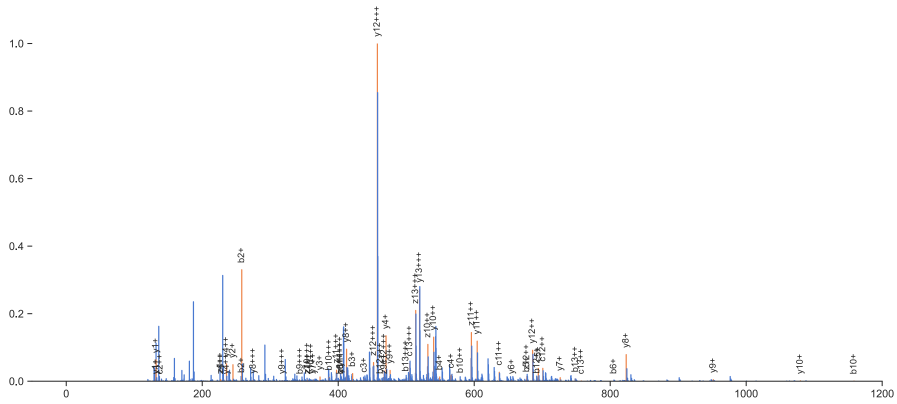

Supplementary Fig. 9 InstaNovo detects peptides from different fragmentation schemes. Spectrum of peptide sequence AWYQQKPGKAPKLL, assigned correctly by Instanovo in the Herceptin light chain. The spectrum was generated with EThcD fragmentation from the elastase digested antibody samples.

Supplementary Tables

Supplementary Table 1 General training setup and model hyperparameters. InstaNovo+ uses the same parameters as InstaNovo unless otherwise specified.

Parameter	*Description*	*Value*
	Training hyperparameters	
`framework`	Python implementation framework	PyTorch Lightning
`optimiser`	PyTorch Lightning model optimiser	Adam
`batch_size`	Optimiser number of samples per batch	32
`learning_rate`	Optimiser learning rate	5e-5
`epochs`	Number of training epochs per experiment	30
`warmup_steps`	Number of LR linear warmup steps	100,000
`n_tune_epochs`	Maximum number of fine-tuning epochs	6
	InstaNovo hyperparameters	
`dim_model`	Hidden dimension of the model	768
`n_head`	Number of attention heads per layer	16
`dim_feedforward`	Feedforward transformer block dimension	1,024
`n_layers`	Number of transformer layers	9
`dropout`	Layer dropout probability	0.1
`isotope_error`	Maximum number of isotopes considered	1
`n_peaks`	Number of peaks pre-filtered	200
`n_beams`	Number of beams in beam-search decoding	5
`max_charge`	Maximum charge considered	10
	InstaNovo+ hyperparameters	
`decoder_layers`	Number of diffusion decoder layers	12
`n_head`	Number of decoder attention heads per layer	8
`T`	Number of diffusion timesteps	20
`dropout`	Layer dropout probability	0.1
	Hardware setup A	
`gpu`	Graphics processing unit	Nvidia RTX 3090ti
`cpu`	Central processing unit	Intel i5-13600k
`vram`	Available GPU virtual memory (for models)	24GB
`ram`	Available system memory (for datasets)	64GB
	Hardware setup B	
`gpu`	Graphics processing unit	Nvidia A100-80GB
`cpu`	Central processing unit	AMD EPYC 7,742 (32 threads used)
`vram`	Available GPU virtual memory (for models)	80GB
`ram`	Available system memory (for datasets)	256GB

Supplementary Table 2 Nanobodies included in this study, including both an original Sanger sequencing round, as well as re-sequencing for sequence confirmation. Discrepancies are highlighted in red.

Sequencing status	*Name*	*Sequence*	*Length*
Original	TPL0612_01_A09	QVQLQESGGGLVEPGGSLRLSCAVSGGSLNHYAMAWFRQAPGQEREGVACINRSGISTTYADSVKGRFTISRDNTKNTVWLQMNSLKPEDTGVYYCSAGKYYTVGHCDQDDYRGQGTQVTVSSAAADYKDHDGDYKDHDIDYKDDDDKGAAHHHHHH	157
Resequenced	TPL0612_01_A09_(+1)	QVQLQESGGGLVEPGGSLRLSCAVSGGSLNHYAMAWFRQAPGQEREGVACINRSGISTTYADSVKGRFTISRDNTKNTVWLQMNSLKPEDTGVYYCSAGKYYTVGHCDQDDYRGQGTQVTVSSAAADYKDHDGDYKDHDIDYKDDDDKGAAHHHHHH	157
Original	TPL0464_01_G08	QVQLQESGGGLVEPGGSLRLSCAASGFTFINYRMSWVRQAPGKGLEWVSGINPDGGTSYSDSVKGRFTISRDNAKNTLYLQMNSLKVEDTAVYYCIQSGTSRRGQGTQVTVSSAAADYKDHDGDYKDHDIDYKDDDDKGAAHHHHHH	147
Resequenced	TPL0464_01_G08_(+1)	QVQLQESGGGLVEPGGSLRLSCAASGFTFINYRMSWVRQAPGKGLEWVSGINPDGGTSYSDSVKGRFTISRDNAKNTLYLQMNSLKVEDTAVYYCIQSGTSRRGQGTQVTVSSAAADYKDHDGDYKDHDIDYKDDDDKGAAHHHHHH	147
Original	TPL0465_01_G08	QVQLQESGGGLVQAGGSLRLSCLASGRTFSDYRIGWFRQAPGKEREFVSTIRNDDANTYYADSVKGRFTISRDNAKNTVYLQMNSLKPEDTAVYYCAAGARHTAQTMAAGKGIDYWGQGTQVTVSSAAADYKDHDGDYKDHDIDYKDDDDKGAAHHHHHH	160
Resequenced	TPL0465_01_G08_(+1)	QVQLQESGGGLVQAGGSLRLSCLASGRTFSDYRIGWFRQAPGKEREFVSTIRNDDANTYYADSVKGRFTISRDNAKNTVYLQMNSLKPEDTAVYYCAAGARHTAQTMAAGKGIDYWGQGTQVTVSSAAADYKDHDGDYKDHDIDYKDDDDKGAAHHHHHH	160
Original	TPL0605_01_G08	QVQLQESGGGLVQAGGSLRLSCAASGRPLRDFAMGWFRQAPGKEREFVAAISSGGSTYYADSMKGRFTISRDNAENTVYLQMNSLKPEDTCVYYCAATMYSAATMSTRRPDYWGQGTQVTVSSAAADYKDHDGDYKDHDIDYKDDDDKGAAHHHHHH	157
Resequenced	TPL0605_01_G08_(+1)	QVQLQESGGGLVQAGGSLRLSCAASGRPLRDFAMGWFRQAPGKEREFVAAISSGGSTYYADSMKGRFTISRDNAENTVYLQMNSLKPEDTCVYYCAATMYSAATMSTRRPDYWGQGTQVTVSSAAADYKDHDGDYKDHDIDYKDDDDKGAAHHHHHH	157
Original	TPL0604_01_E02	QVQLQESGGGLVQAGGSLRLSCAASGRTFDDYSMGWFRQAPGKEYEFVASINWSGSYTYYTDSVKGRFTISRDNAKNTVYLQMNSLKPDDTAVYYCAARDSIGVAVRRIDYDYWGQGTQVTVSSAAADYKDHDGDYKDHDIDYKDDDDKGAAHHHHHH	158
Resequenced	TPL0604_01_E02_(+1)	QVQLQESGGGLVQAGGSLRLSCAASGRTFDDYSMGWFRQAPGKEYEFVASINWSGSYTYYTDSVKGRFTISRDNAKNTVYLQMNSLKPDDTAVYYCAARDSIGVAVRRIDYDYWGQGTQVTVSSAAADYKDHDGDYKDHDIDYKDDDDKGAAHHHHHH	158
Original	TPL0611_01_C09	QVQLQESGGGLVQPGGSLRLSCAASGNIFSINYMKWYRQAPGKQRELVAVITDGGRTNYADSVKGRFAISRDNAKNTTYLQMSDLQPEDTAVYYCYADLRVVDGRHLPRGDYWGQGTQVTVSSAAADYKDHDGDYKDHDIDYKDDDDKGAAHHHHHH	157
Resequenced	TPL0611_01_C09_(+1)	QVQLQESGGGLVQAGGSLRLSCAASGRTFSMRNMGWFRQAPGKEREIVATISRSGGSTDYGDSVKGRFTISTDNAKNTAYLLMNSLKPEDTAVYYCAADLFGTRQADLLIYNFRGQGTQVTVSSAAADYKDHDGDYKDHDIDYKDDDDKGAAHHHHHH	158
Resequenced	TPL0611_01_C09_re-sequenced_(+1)	QVQLQESGGGLVQAGGSLRLSCAASGRTFSMRNMGWFRQAPGKEREIVATISRSGGSTDYGDSVKGRFTISTDNAKNTEYLLMNSLKPEDTAVYYCAADLFGTRQADLLIYNFRGQGTQVTVSSAAADYKDHDGDYKDHDIDYKDDDDKGAAHHHHHH	158
Original	TPL0436_01_G05	QVQLQESGGGLVQAGGSLRLSCAASGRTFSRYTMGWFRQASGKEREFVAAIRWPGGNTYYSDSVKGRFTISTTISTNNAKNTLYLQMNSLQPEDTAVYYCAAVRGTFFDGGDYINHHQYDYWGQGTQVTVSSAAADYKDHDGDYKDHDIDYKDDDDKGAAHHHHHH	166
Resequenced	TPL0436_01_G05_(+1)	QVQLQESGGGLVQAGGSLRLSCAASGRTFSRYTMGWFRQASGKEREFVAAIRWPGGNTYYSDSVKGRFTISTTISTNNAKNTLYLQMNSLQPEDTAVYYCAAVRGTFFDGGDYINHHQYDYWGQGTQVTVSSAAADYKDHDGDYKDHDIDYKDDDDKGAAHHHHHH	166
Original	TPL0615_01_E01	QVQLQESGGGLVQAGGSLRLSCAASGRTFSSYAMAWFRQAPGKEREFVASISWSGDSTYYADSVKGRFTISRDNAKNTWYLQMNSLKPEDTAVYYCNTEEESTGTYYEWGQGTQVTVSSAAADYKDHDGDYKDHDIDYKDDDDKGAAHHHHHH	153
Resequenced	TPL0615_01_E01_(+1)	QVQLQESGGGLVQAGGSLRLSCAASGRTFSSYAMAWFRQAPGKEREFVASISWSGDSTYYADSVKGRFTISRDNAKNTWYLQMNSLKPEDTAVYYCNTEEESTGTYYEWGQGTQVTVSSAAADYKDHDGDYKDHDIDYKDDDDKGAAHHHHHH	153
Original	TPL0471_01_B06	QVQLQESGGGLVQPGGSLRLSCTASLNIFSINAMGWYRQAPGKQRELVAAITSGGSTNYADSVKGRFTISRDNAKSTVYLQMNSLKPEDTAVYYCHAEGPFNIATKEQYDYWGQGTQVTVSSAAADYKDHDGDYKDHDIDYKDDDDKGAAHHHHHH	156
Resequenced	TPL0471_01_B06_(+1)	QVQLQESGGGLVQPGGSLRLSCTASLNIFSINAMGWYRQAPGKQRELVAAITSGGSTNYADSVKGRFTISRDNAKSTVYLQMNSLKPEDTAVYYCHAEGPFNIATKEQYDYWGQGTQVTVSSAAADYKDHDGDYKDHDIDYKDDDDKGAAHHHHHH	156
Original	TPL0464_01_E02	QVQLQESGGGLVQPGGSLRLSCAASGFTVSSVTLSWLRQAPGKGLEWVSDITSNGQTYYADSVKGRFTISRDNAKNTIYLQMNSLKADDSAVYFCAEDRWRSSNHPRGQGTQVTVSSAAADYKDHDGDYKDHDIDYKDDDDKGAAHHHHHH	151
Resequenced	TPL0464_01_E02_(+1)	QVQLQESGGGLVQPGGSLRLSCAASGFTVSSVTLSWLRQAPGKGLEWVSDITSNGQTYYADSVKGRFTISRDNAKNTIYLQMNSLKADDSAVYFCAEDRWRSSNHPRGQGTQVTVSSAAADYKDHDGDYKDHDIDYKDDDDKGAAHHHHHH	151
Original	TPL0599_01_G01	QVQLQESGGGLVQPGGSLRLSCAASGSASSMYTLAWYRQAPGKQRELVALITSGHMTHYEDSVKGRFTISRDNAKEVLYLQMNSLKPEDTAVYFCNLHRLTSSDDDGRTWGQGTQVTVSSAAADYKDHDGDYKDHDIDYKDDDDKGAAHHHHHH	154
Resequenced	TPL0599_01_G01_(+1)	QVQLQESGGGLVQPGGSLRLSCAASGSASSMYTLAWYRQAPGKQRELVALITSGHMTHYEDSVKGRFTISRDNAKEVLYLQMNSLKPEDTAVYFCNLHRLTSSDDDGRTWGQGTQVTVSSAAADYKDHDGDYKDHDIDYKDDDDKGAAHHHHHH	154
Original	TPL0431_01_A07	QVQLQESGGGLVQPGGSLRLSCAASGSIVNINYMRWYRQAPGKQRELVAVITSAGNTNYAESVKGRFTISIDNAKKMVFLQMNSLKPDDTAVYYCHADLRVRDGVRGDYWGQGTQVTVSSAAADYKDHDGDYKDHDIDYKDDDDKGAAHHHHHH	154
Resequenced	TPL0431_01_A07_(+1)	QVQLQESGGGLVQPGGSLRLSCAASGSIVNINYMRWYRQAPGKQRELVAVITSAGNTNYAESVKGRFTISIDNAKKMVFLQMNSLKPDDTAVYYCHADLRVRDGVRGDYWGQGTQVTVSSAAADYKDHDGDYKDHDIDYKDDDDKGAAHHHHHH	154
Original	TPL0604_01_D12	QVQLQESGGGLVQPGGSLRLSCAASGVTLDYYGTGWFRQAPGKEREGVACIRSSDGSTYYADSVKGRFTISRDNAKNMVYLQMNSLKPEDTAVYYCAAERVPSAIYTHYEFCVGADEYDYWGQGTQVTVSSAAADYKDHDGDYKDHDIDYKDDDDKGAAHHHHHH	165
Resequenced	TPL0604_01_D12_(+1)	QVQLQESGGGLVQPGGSLRLSCAASGVTLDYYGTGWFRQAPGKEREGVACIRSSDGSTYYADSVKGRFTISRDNAKNMVYLQMNSLKPEDTAVYYCAAERVPSAIYTHYEFCVGADEYDYWGQGTQVTVSSAAADYKDHDGDYKDHDIDYKDDDDKGAAHHHHHH	165

Supplementary Table 3 Primers used in this study for *E. coli* and *P. aeruginosa*.

Organism	*Direction*	*Sequence*
E. coli	Forward	GGAAGAAGCTTGCTTCTTTGCTGAC
E. coli	Reverse	AGCCCGGGGATTTCACATCTGACTTA
P. aeruginosa	Forward	GACGGGTGAGTAATGCCTA
P. aeruginosa	Reverse	CACTGGTGTTCCTTCCTATA

Supplementary Notes

SI Note 1: Additional dataset details

The processed data, detailed in Section 4.1.1, were organised into individual data frames for each run, and subsequently merged to create a consolidated dataset for model training. The key features incorporated into the dataset included mass values, intensity, "MS/MS m/z," charge, and modified sequence. The same procedure was followed for testing and validating the model. In cases where additional validation was required on all MS2 spectra recorded for each data acquisition run, a separate validation dataset was created. This dataset included all of the MS2 spectra from the raw files (selecting the 800 most intense peaks from each spectrum if more were present), as well as associated metadata, i.e. charge, precursor mass, retention time, and measurement error.

External dataset retrieval and processing

External datasets were also processed using the same methodology as with the training dataset. Publicly available and in house datasets were used to benchmark and validate the model. Specifically, the antibody dataset [1] was used for benchmarking against similar *de novo* peptide sequencing approaches. The immunopeptidomic dataset [2], used for validation of the model in HLA peptide performance and efficiency, can be found in the PRIDE repository with identifier PXD006939. The snake venom dataset was downloaded from this article [3] and can be found in the PRIDE repository with identifier PXD036161. The wound exudates originated from this article [4] and are available in PanoramaWeb with dataset identifier PXD025748. The herceptin dataset was found in a *de novo* sequencing tool comparison study [1] in Figshare (doi.org/10.6084/m9.figshare.21394143) [5].

SI Note 2: Additional model details

InstaNovo

We use multi-scale sinusoidal embeddings [6] to encode our peaks. In these encodings, the m/z peaks are encoded with varying frequencies along the hidden dimension of the encoded output. These encodings are then processed with two dense layers, concatenated with the intensity vector, and another two dense layers – providing a high quality encoding of the spectral information. We have confirmed that this approach matches and surpasses other embedding approaches. Hence, we have included this method of latent representation of mass spectra in our model.

For a sequence of residues $y_{1:M}$ InstaNovo assigns the probability

$$p(y_{1:M}|\mathbf{m}, \mathbf{I}, m_{prec}, c_{prec}) = \prod_{i=1}^{M} p(y_i|y_{<i}, \mathbf{m}, \mathbf{I}, m_{prec}, c_{prec})$$

$$= \prod_{i=1}^{M} p(y_i|\mathsf{dec}(y_{<i}, \mathsf{enc}(\mathbf{m}, \mathbf{I}, m_{prec}, c_{prec})))$$

where $\mathsf{enc}(\mathbf{m}, \mathbf{I}, m_{prec}, c_{prec})$ are the embeddings returned by feeding the spectra, precursor mass, and precursor charge into a transformer encoder, as described in Section 4.2.1, and $\mathsf{dec}(y_{<i}, \mathbf{x})$ are the embeddings returned by a transformer decoder fed sequence $y_{<i}$ and cross-attending on tensor $\mathbf{x}$. $p(y_i|\mathbf{v})$ is parameterised as a linear layer followed by a softmax, and we perform training and inference using log-probabilities for numerical stability. Our system also returns the sequence of per-residue log-probabilities $\log p(y_i|y_{<i}, \mathbf{m}, \mathbf{I}, m_{prec}, c_{prec})$ for quantifying uncertainty around predicted residues in downstream analysis.

InstaNovo+

Since InstaNovo is auto-regressive, there is no typical approach to correcting errors made early on during decoding. This is particularly problematic due to the peptide fragmentation properties. The first fragmentation products of a peptide might not even be present in the spectrum due to the first mass cutoff in the spectrum acquisition, or they might not behave ideally in the mass spectrometer due to their physicochemical properties. Beam-search would ideally remedy this, but as the sequence length increases, the issue remains.

Similarly, we thought that starting residue prediction or sequence order might be wrong, especially in cases where fragment ions are not that intense or spectrum landscapes are noisy. In such cases, the model does not have a chance to update its prediction, or take into account multiple series of ions. Hence, as detailed in Section 4.2.2, we propose InstaNovo+. InstaNovo+ has 170M parameters in total.

To perform multinomial diffusion, we define three distributions applied to a discrete fixed-length sequence $\mathbf{x}_t$, where t is the current noise step. $\mathbf{x}_0$ represents an uncorrupted peptide and $\mathbf{x}_T$ is a completely corrupted peptide indistinguishable from the starting point $\mathbf{x}_0$. T is a hyper-parameter representing the maximum number of noising steps, and is chosen as 20 in this work.

We can now define the diffusion processes. Firstly, the forward noising function $q(\mathbf{x}_t \mid \mathbf{x}_{t-1})$ defines the distribution over $\mathbf{x}_t$ given $\mathbf{x}_{t-1}$. Secondly, the denoising function $q(\mathbf{x}_{t-1} \mid \mathbf{x}_t, \mathbf{x}_0)$ defines the distribution over $\mathbf{x}_{t-1}$ given $\mathbf{x}_t$ and $\mathbf{x}_0$. Finally the denoising function $p(\mathbf{x}_{t-1} \mid \mathbf{x}_t)$ defines the distribution over $\mathbf{x}_{t-1}$ conditioned on $\mathbf{x}_t$ and the model output $\hat{\mathbf{x}}_0$:

$$
\begin{aligned}
q(\mathbf{x}_t \mid \mathbf{x}_{t-1}) &= \mathcal{C}\left(\mathbf{x}_t\,;\;\; (1-\beta_t)\mathbf{x}_{t-1} + \beta_t/K\right) \\
q(\mathbf{x}_{t-1} \mid \mathbf{x}_t, \mathbf{x}_0) &= \mathcal{C}\left(\mathbf{x}_{t-1}\,;\; \frac{1}{A}\left[\alpha_t \mathbf{x}_t + (1-\alpha_t)/K\right] \odot \left[\bar{\alpha}_{t-1}\mathbf{x}_0 + (1-\bar{\alpha}_{t-1})/K\right]\right) \\
p(\mathbf{x}_{t-1} \mid \mathbf{x}_t) &= \mathcal{C}\left(\mathbf{x}_{t-1}\,;\; \frac{1}{A}\left[\alpha_t \mathbf{x}_t + (1-\alpha_t)/K\right] \odot \left[\bar{\alpha}_{t-1}\hat{\mathbf{x}}_0 + (1-\bar{\alpha}_{t-1})/K\right]\right)
\end{aligned}
$$

Where $\mathcal{C}$ denotes a categorical distribution. β_t is the noise schedule, $\alpha_t = 1 - \beta_t$, and $\alpha_t = \prod_{\tau=0}^{t} \alpha_\tau$. The noise schedule α_t follows a cosine decay from 1 to 0 as t increases [7]. 1/A denotes a normalising constant. The model predicts $\hat{\mathbf{x}}_0$ given the spectra, precursor information, the current timestep, and the corrupted sequence $\mathbf{x}_t$. To train the model, we compute the KL-divergence between the true denoising distribution $q(\mathbf{x}_{t-1} \mid \mathbf{x}_t, \mathbf{x}_0)$ and the model's distribution $p(\mathbf{x}_{t-1} \mid \mathbf{x}_t)$. Once trained, we can iteratively refine a sequence starting from random noise to arrive at a final prediction for the spectra. Alternatively, we can refine a prediction made by IN, which yielded the best results. To do this, we start from the IN prediction at $t = 15$, and iteratively refine till we reach $\mathbf{x}_0$. This serves as our IN+ prediction. We can also estimate the log probability of IN+ predictions to extract model confidence. We sample a few $\mathbf{x}_t$ given the model prediction as $\mathbf{x}_0$, then take the average KL-divergence loss of the model predicting $\hat{\mathbf{x}}_0$ given $\mathbf{x}_t$. This serves as a lower-bound estimate of the log probability.

SI Note 3: Analysis rationale

During dataset preprocessing, we filter out spectra with more than 800 peaks. In our labelled datasets, we remove PSMs with more than 30 residues length. Both of these constraints were set due to the necessity for determining a maximum number of peaks and maximum number of residues in peptide sequences for the input and output vector sizes of our models respectively. These cutoffs were selected based on observed distributions of these attributes in the AC-PT dataset from Proteome Tools, although the dataset consisted of pools of synthetic peptides. We are aware that by setting these thresholds, we are missing a significant number of spectra and PSMs from our biological datasets, as well as any potential testing datasets to come. Especially in data acquisition runs where the spectra have higher noise, i.e. contains higher interference and contaminants, or in cases where MS2 spectra are acquired in profile mode, the strict cutoff of 800 peaks might be too low. To alleviate these issues in future studies, we are planning on selecting the top 800 peaks from each MS2 spectrum, instead of completely removing them from the dataset (as we did in this study for the full search space datasets). A prime example of this noisiness was observed in the immunopeptidomics dataset reanalysed in this study, where only 651 spectra passed this peak threshold. Therefore, only the full database search results and the prediction for all scans in that dataset was used for

downstream analysis. In the future, a model with double the number of maximum output residues (60 instead of 30) is envisioned, which will cover the vast majority of bottom-up applications, and a larger model can be considered for top down applications if deemed possible.

When reporting protein numbers, unless otherwise specified, we talk about protein groups, meaning the number of candidate groups that peptides map to, or the set of the first protein identifiers for each peptide mapping. Protein inference, grouping, and master protein assignment was out of the scope of this study.

We regard the residues isoleucine and leucine as interchangeable when inferring and mapping predicted sequences to proteins, as these amino acids are isobaric. Although it might be possible through the detection of w ions for the model to distinguish between the two isobaric amino acids, this differentiation is not yet possible with our model and fine-tuning or improving on this will be part of future efforts. Protein coverage was calculated as the number of amino acids covered by PSMs or predictions, by non-redundant peptide sequences, expressed as a percentage.

To evaluate FDR in our *de novo* peptide sequencing predictions, we ground our model's confidence with the FDR estimation of the associated database searches. This means that we extract the scans that have a PSM in the database search, and compare these with our predictions for the same scans, ranked based on model confidence. Once we have reached a 5% FDR threshold, as decided by the PSM-prediction comparison (in this case, the database search is our ground truth, and the PSMs in it our correct labels), we regard this confidence threshold as our model's threshold for 5% FDR. We are aware of the limitations of this approach, and are working on better assessment criteria for FDR in *de novo* peptide sequencing.

SI Note 4: Metric formulations

For each spectrum, a model decodes a peptide with some confidence. For a given confidence threshold, the model predicts peptides for spectra where this confidence is higher than the threshold and for other spectra it predicts nothing. An amino acid in a model-predicted peptide matches one in a gold standard peptide if their masses differ by less than 0.1 Da and the masses of their prefixes differ by less than 0.5 Da. A model-predicted peptide matches a gold standard peptide if they have the same number of amino acids and every amino acid in the model-predicted peptide matches one in the gold standard peptide. Amino acid precision and recall are defined as $N^A_{\text{match}}/N^A_{\text{pred}}$ and $N^A_{\text{match}}/N^A_{\text{gold}}$ respectively, where N^A_{match} is the total number of matched amino acids in model-predicted peptides, N^A_{pred} is the total number of amino acids in model-predicted peptides and N^A_{gold} is the total number of amino acids in gold standard peptides. Peptide precision and recall are defined as $N^P_{\text{match}}/N^P_{\text{pred}}$ and $N^P_{\text{match}}/N^P_{\text{gold}}$ respectively where N^P_{match} is the total number of matched model-predicted peptides, N^P_{pred} is the total number

of model-predicted peptides and N^P_{gold} is the total number of gold standard peptides.

SI Note 5: Beam search with knapsack filtering

Finding all amino acid sequences whose theoretical mass is in some range is an instance of the knapsack problem and can be solved efficiently using dynamic programming or graph search over an array of possible masses called a *chart*. We pre-computed this chart for masses up to 4,000 Da at a resolution of 0.0001 Da using depth-first search (Supplementary Note 5, Algorithm 2). During beam search, we used the chart to filter out sequences that could not be continued to fit the precursor mass to the tolerance. As a result, all partial sequences on the beam could be continued to fit the precursor mass guaranteeing the system could always find a peptide that matched the precursor to the tolerance.

Knapsack chart generation algorithm

Algorithm 1 Knapsack chart generation by DFS

Input: M the maximum mass, $\mathcal{R}$ the set of residues
Output: $chart$, an $(M+1)\times \mid \mathcal{R} \mid$ boolean array where $chart[m, r]$ is true if and only if there exists a mass m sequence starting with residue r

```
visited ← {}                                        ▷ Visited set and chart initialization
chart[0 : M, R] ← False

for residue ∈ R do                                  ▷ Initial chart population.
    chart[getMass(residue), residue] ← True
    stack.push(getMass(residue))
end for

while ¬stack.empty do                               ▷ Recursive chart population.
    mass ← stack.pop()
    if mass ∈ visited then
        continue
    end if
    for residue ∈ R do
        if mass + getMass(residue) ≤ M then
            chart[mass + getMass(residue), residue] ← True
            stack.push(mass + getMass(residue))
        end if
    end for
    visited.add(mass)
end whilereturn chart
```

Beam Search decoding with knapsack filtering algorithm

Algorithm 2 Beam Search decoding with knapsack filtering

Input: $\mathbf{S} = (\mathbf{m}_1, I_1), \cdots, (\mathbf{m}_n, I_n)$ a spectrum consisting of mass-intensity values, $\mathcal{R}$ a set of residues, δ_m a mass tolerance, k a beam width
Output: $P = R_1, \cdots, R_k$ a peptide consisting of residues R_i from $\mathcal{R}$

$\mathbf{z} \leftarrow \textsc{Encoder}(\mathbf{S}, m_{pre}, q_{pre})$ ▷ Precompute spectrum representations

$\mathcal{C} \leftarrow \{\}$ ▷ Initialize set of complete sequences
$\mathcal{B} \leftarrow \{\langle \epsilon, m_{pre}, 0 \rangle\}$ ▷ Initialize beam

for $i \leftarrow 1, \cdots, L$ **do**
  $\mathcal{B}' \leftarrow \{\}$
  for $x, m, \log p(x \mid \mathbf{z}) \leftarrow \mathcal{B}$ **do** ▷ Expand and score candidate residues
    for $r \leftarrow \mathcal{R}$ **do**
      $m' \leftarrow m - \mathsf{getMass}(r)$
      $\log p(x \cdot r \mid \mathbf{z}) \leftarrow \log p(r \mid \textsc{Decoder}(x, \mathbf{z})) + \log p(x \mid \mathbf{z})$
      if $\mid m' \mid \leq \delta_m$ **then**
        $\log p(x \cdot r \cdot \$ \mid \mathbf{z}) \leftarrow \log p(\$ \mid \textsc{Decoder}(x \cdot r, \mathbf{z})) + \log p(x \cdot r \mid \mathbf{z})$
        $\mathcal{C}.\mathsf{push}(\langle x \cdot r, \log p(x \cdot r \cdot \$ \mid \mathbf{z}) \rangle)$
      else if $\textsc{Any}(chart[m - \delta_m : m + \delta_m, r])$ **then**
        $\mathcal{B}'.\mathsf{push}(\langle x \cdot r, m', \log p(x \cdot r \mid \mathbf{z}) \rangle)$
      end if
    end for
  end for
  $\mathcal{B} \leftarrow \textsc{Top-}k(\mathcal{B}', k)$ ▷ Prune and update beam
end forreturn $\textsc{Top}(\mathcal{C})$ ▷ Return highest scoring complete sequence

SI Note 6: InstaNovo inference speeds

We evaluated inference of our models and found that the IN architecture predicts a peptide sequence with an average 142 ms / spectrum, or 120 minutes for a typical single shot MS run of 50 k spectra. IN+ processes the same number of spectra in 26 minutes, with batch sizes of 64 spectra processed in 1.94s (performance measured on a consumer-level hardware: Intel i5-13600k CPU, RTX 3090ti GPU). When using InstaNovo predictions as a starting point for InstaNovo+, the total runtime would be the sum of the two model runtimes. The model runtime scales linearly with the number of spectra, which is invariant in terms of protein digestion, closed or open searches, type of fragmentation or resolution, or number of modifications (Supplementary Fig. 6). These numbers compare well with search times for conventional proteomics analysis platforms with database search implementations. We expect clear improvement in runtime and PSM detection rates in datasets searched against large protein databases or datasets with a large amount of spectra.

SI Note 7: Further comparative performance evaluation

We conducted further performance evaluations of IN against Casanovo [8]. We again utilised the high-resolution nine-species dataset [9], and the ProteomeTools [10] dataset.

On the nine-species dataset, we evaluated the peptide-level model accuracy in three setups (Fig. 2d and Supplementary Fig. 3 a and b). Firstly, in Supplementary Fig. 3 b, the models were trained on nine-species excluding the holdout species, and then evaluated on the holdout species. In this setup, the dataset was too small to effectively train IN and IN+, as the model architectures were designed for larger datasets. Hence, we exclude IN+ from this comparison, and see significantly worse performance on IN in this setup compared to IN when trained on the HC-PT dataset. Secondly, in Supplementary Fig. 3a, we compared the models trained on HC-PT and evaluated directly on the holdout species. In this case, we found IN+ performed the best, outperforming Casanovo across all holdout species, validating our choice of architecture. Lastly, in Fig. 2d, we compared the models trained on HC-PT and then fine-tuned on the nine-species excluding the respective holdout species. All three models improved significantly, with IN+ reaching a peptide-level accuracy of 62.36%, 67.44%, and 49.04%, on yeast, bacillus and mouse, respectively. We also assessed the accuracies of each model on HC-PT and AC-PT, grouped by tryptic, HLA-I, HLA-II, LysN, and AspN peptides (Supplementary Fig. 4 a and b). We observe robust performance in tryptic and peptides associated with HLA class I, while accuracy is diminished in peptides associated with HLA class II and peptides generated with alterative proteases.

Casanovo implementation

We compared our model to a state-of-the-art deep learning method that has been shown to have superior performance, has been replicated and their architecture is of interest, this being Casanovo [8]. Casanovo frames the problem as a sequence-to-sequence problem [8] and employs a transformer encoder-decoder framework to process and predict sequences of amino acids. Casanovo was retrained using their official GitHub repository (github.com/Noble-Lab/casanovo). The model was trained for 30 epochs, considering a total of 12 ion types with a maximum peptide length of 60 and peptide mass of 5,000 Da. The maximum number of peaks per spectrum was set at 400. Fixed modifications for each dataset included carbamidomethylation of cysteine (C + 57.02 Da). Variable modifications for all datasets were oxidation of methionine (M + 15.99 Da), deamidation of asparagine (N + 0.98 Da) and glutamine (Q + 0.98 Da). It is important to note that only carbamidomethylation and oxidation of methionine were observed in the dataset analysis of the Prosit dataset.

SI Note 8: Additional robust performance results of InstaNovo

In addition to the results detailed in Section 2.3, we observed multiple instances of our models predicting sequences from spectra with high confidence, while database searches matched the same spectra to different sequences. In some of those cases, analysis with spectrum similarity correlation metrics indicates that our model predictions match the experimental spectrum better than the database search results (Supplementary Fig. 7). This suggested that the model can improve the precision of database search PSMs if applied in the context of an auxiliary or re-scoring search engine in database searches. Importantly, we further monitored predictions of novel peptides with targeted proteomics experiments, validating our model experimentally (Supplementary Fig. 8). These results demonstrate that the model predicts *bona fide* peptide sequences without any prior information, and can inherently discern between good and bad predictions with high fidelity based on the prediction model confidence.

SI Note 9: Additional details on the evaluations of InstaNovo on application-focused datasets

Nanobody production

Camelids were immunised with whole venoms from either 8 viperid snake species or 18 elapid snake species, followed by the construction of immune nanobody displaying phage libraries (VIB Nanobody Core, Brussels) as described by Pardon et al. [11]. These libraries were used in phage display selection campaigns including purified biotinylated snake toxins as antigens in a procedure similar to the one described by Ledsgaard et al. [12] with the exception that the nanobody-encoding genes were digested with *Pst1* and *Eco91l* restriction enzymes and subcloned into the xb-145 vector for expression

(instead of using *NcoI* and *NotI* restriction enzymes and the pSANG10-3F vector). Individual colonies were picked, cultivated, and used for expression of nanobodies. The periplasmic fractions of the cells (expected to contain the majority of the nanobody protein) were analysed in an expression-normalised capture DELFIA [13]. A subset of nanobodies binding their cognate antigens were Sanger sequenced (Eurofins Genomics) using the M13rev-29 primer. For purification of the nanobodies, *E. coli* BL21(DE) were grown at 37 °C and 220 rpm until OD_{600} reached 0.5, followed by the addition of 0.5 mM IPTG to induce protein expression. After 16 h incubation at 30 °C and 220 rpm, the cells were collected by centrifugation at 4,000 g for 15 minutes at 4 °C and stored at -20 °C. Thereafter, the frozen cells were resuspended in ice-cold PBS supplemented with 10 mM imidazole and an EDTA-free protease inhibitor cocktail (Roche). Supernatants containing the nanobodies were collected by centrifugation at 20,000 g for 45 minutes at 4 °C. The His-tagged nanobodies were captured on an Ni^{2+}-NTA affinity resin (Thermo Fisher Scientific) by gravity flow. Unbound proteins were washed away, and the nanobodies were eluted (PBS with 200 mM NaCl and 250 mM imidazole), after which the imidazole was removed by dialysis.

InstaNovo increases PSM rate in HeLa proteomes

First, we wanted to investigate the performance of IN in one of the gold standards in proteomics experiments, the lysate of HeLa cells. HeLa proteomes are frequently used for benchmark studies, and are also routinely used as quality controls between sample batches during data acquisition to assess instrument performance and robustness. We analysed 200 ng of HeLa cells with an Orbitrap Exploris 480 mass spectrometer. IN was able to obtain a 49.6% recall in the HeLa single-shot dataset, assigning correct (identical to the database search) sequences for 8,774 PSMs. At 99.9% confidence, we observe 1% false positives, as assessed by grounding to the database search. At 98.1%, we observe 5% false positive hits, indicating that model confidence sufficiently captures prediction certainty, and suggesting optimal model thresholds that might be used for relevant FDR cutoffs. With a confidence cutoff equivalent to 5% FDR for sequence predictions, IN increased the database search PSM identification rate by 7.5%, identifying 1,338 more PSMs in the MS/MS scans that did not result in any database search hits. Using IN+, we detected 365 more correct PSMs from database searches, even though the total PSM rate did not improve for this dataset. Using the average of output amino acid confidences for each prediction, generating 5 predictions for each spectrum and filtering for sequences that match the precursor mass, we observed in most cases (n=11,979, 87,4% of subset where any prediction matches the precursor mass, 67,74% of the complete PSM space) the first prediction matches the precursor mass, suggesting that the model outputs the correct sequence with the prediction possessing the highest confidence score, where possible (Fig. 4a). Next, we wondered whether our additional predicted sequences would match to the human proteome without any prior database information, and whether

the protein coverage and PSM rate would be increased. By choosing a cutoff of 98% prediction confidence, equating to 5% FDR for IN (Fig. 4b) and performing similar thresholding for IN+ (Fig. 4c), we could assess model predictions at a low FDR rate, as per standard practice in proteomics experiments. Considering only IN predictions that match the precursor mass, we were able to naively match 4,029 PSMs corresponding to 3,836 unique peptide sequences, mapping to 1,595 proteins in the human database. It is worth noting that prediction of longer sequences that map to a standard reference proteome, even at lower confidence thresholds, is a direct way of obtaining matches with low FDRs. The chances of obtaining a hit at random with a peptide sequence of 6 residues, usually the lowest peptide length allowed in mass spectrometry, is approximately 1 in 1,600. When considering a length of 10 residues (the mode of peptide length observed experimentally), the number approaches 1 in 4 billion of getting a hit by chance. These approximations were also what we observed in simulations with randomly generated databases of equal size to the human proteome and the same prediction confidence threshold, where the few random matches encountered were 6-8 residues long (Extended Fig. 4a), different in length distribution compared to the predicted sequences, and significantly lower than the PSM rate observed in the human database (two-sided one sample t-test, p-value=$3.9e^{-26}$). The few random matches to these generated databases from our model predictions were obtained for database search PSMs at the lower end of the search engine's score and posterior error probability, validating once again our model and its confidence as a suitable metric for FDR estimation. For our predicted human proteome matches, we could observe that the mode of the peptides per protein distribution is 1, and follow a hypergeometric distribution, similar to PSMs or peptides per protein distribution of database searches in bottom-up proteomics (Extended Fig. 4b and c). Similarly, the peptide length distribution is in agreement with the database search results. IN also predicted peptide sequences with 1 or more missed cleavages, in the same relative amount as database searches, indicating an adequate performance on peptides that contain internal lysine or arginine residues (Extended Fig. 4d). Satisfied with the performance of our base model, we evaluated our knapsack algorithm for selection of model outputs. With a 74% confidence threshold in our knapsack predictions, which are multiplied amino acid probabilities across the peptide sequence (corresponding to 5% FDR), we detected 4,527 PSMs mapping to 4,308 unique peptide sequences and 1,729 proteins. Given the superior performance of our knapsack predictions, we henceforth evaluated the models and report results with our knapsack implementation. When analysing the full search space, without any restrictions to the database search results, we obtained similar prediction confidence, peptide length and protein coverage distribution. However, by utilising the full search space (all MS2 spectra), and using identical confidence filters, we are able to retrieve 6,105 predicted PSMs (Fig. 4d), mapping to 5,287 unique peptides and 2,214 proteins in our protein database. Among these, we observed 811 more unique peptides and 151 more proteins that match to the human

proteome, and would otherwise go undetected by database searches (Extended Fig. 4e and f). These numbers correspond to a 29.09% and 20.01% detection rate increase on peptide and protein level, respectively, comparing to the restricted database search space predictions when applying these filters, and a 3.84% and 3.63% peptide and protein identification increase respectively when compared directly to database search results, with no post-prediction filtering and with strict FDR thresholds. We observed gains and new peptide matches in new proteins as well as proteins with previously sequenced peptides, increasing coverage at all levels. Importantly, we noticed several examples of peptides in the database search space where the model predicted sequence theoretical fragmentation spectrum, whether binary or Prosit predicted, correlated better with the experimental spectrum than the database search PSM sequence (Fig. 4e). Using the same cutoffs, we also obtained 442 PSMs with 419 unique peptide sequences that do not map to any human protein. We used protein Blast to determine the origin of those sequences, with the highest confidence sequences corresponding to lysyl endopeptidase, which was used in digestion of the HeLa proteome along with trypsin. Other sequences map to proteins in the human proteome with a single mismatch of asparagine to glutamate residue, a common modification in proteomics experiments. These results suggest that IN generates high confidence predictions which support and expand database search results even in the most comprehensively characterised proteomes.

InstaNovo can sequence engineered biomolecules with near total coverage in a single run

Next, we aimed to examine our model's performance in *de novo* sequencing of novel, engineered biomolecules. To do this, we first expressed and purified 13 nanobody scaffolds raised against different snake toxin antigens with phage display technology, then sequenced them and analysed them with mass spectrometry, using a standard sample preparation workflow with trypsin digestion. As the nanobodies were expressed in E. coli, we ran our database search with the E. coli reference proteome and the 13 nanobody sequences as our background database. In our database search, we detected all 13 nanobodies in our samples with 4,465 PSMs, and sequenced them with 91% average protein coverage, 7 of which with 100% sequence coverage. Out of all proteins detected, we achieved 44.15% peptide recall with IN when querying the database PSMs. When applying IN to the sequencing of our nanobodies with 92% confidence threshold (expected FDR of 5%) in our database search space, we obtained an average protein coverage of 68.93% (13.46% standard deviation), with a median of 16 peptides per protein, and 5 unique peptides per nanobody. When evaluating IN on the database search PSM associated scans, we find 4,955 PSM predictions that match our nanobody sequences with 94 unique peptides (Fig. 4f). This increased detection rate could be mapped to an average of 91 (standard deviation of 13.7) peptides per nanobody, improving our coverage to 91.39% (4.66% standard deviation), reflecting a near 23% increase in

sequence coverage compared to the database search space (Fig. 4g). Importantly, we obtained 7,536 matches mapping to 613 peptides when expanding the search to the full search space (all MS/MS spectra) of our runs, a 6-fold peptide detection increase compared to the PSM space from database searches (Fig. 4h). The unique peptide sequences detected for a nanobody increased to 40, a notable 8-fold increase in average unique sequences when compared to the database search space. These surprising results can be for the most part attributed to semi-tryptic or non-tryptic peptides, products of aminopeptidase activity or degradation, which are distinguished by the ragging patterns of their sequences that differ by one terminal residue at a time. These peptides would be missed in traditional database searches, which limit the computational search space by only considering fully tryptic peptides for the theoretical digest and peptide spectrum scoring. Among the novel peptides discovered in the full search space, there were four peptides mapping to a region of a nanobody with ambiguous genomic sequencing results (C09), elucidating the sequence of that region and demonstrating its promise in similar applications (Extended Fig. 6). IN predicted 5,068 novel PSMs at the same confidence threshold in the full search space, a 24.83% increase in PSM detection rate compared to the database search. IN+ detected 2,016 novel PSMs at 5% FDR, whilst slightly increasing the overall peptide recall.

Additionally, we applied our model to a publicly available dataset evaluating mass spectrometry based antibody sequencing [1]. For this, the authors used 9 different proteases and 2 fragmentation activation types to sequence herceptin (commercially available as Trastuzumab), a monoclonal humanised antibody used to treat breast and stomach cancer by binding to the HER2 receptor [14, 15]. The reason for this was to evaluate our model in a different antibody format prediction, as well as to assess model performance in prediction of peptides generated with several different proteases and fragmentation schemes. Combining the database searches for a subset of 6 out of the 9 different proteases, we detected 1,796 PSMs mapping to 129 unique peptides in the heavy and light chain of herceptin, covering 63.02% of the heavy chain with 83 peptides, and 71.96% of the light chain with 46 peptides. IN achieved 68.99% peptide recall, while it expands detected sequences by 575 unique peptide sequences at 5% FDR, obtaining similar detection rate increases to our nanobody sequencing results. Importantly, it increases protein coverage to 92.87% and 100% for heavy and light chains, respectively (Fig. 4i). Interestingly, IN assigns the correct sequence for PSMs generated by all proteases tested (27.63% PSM recall), with lowest success rates for LysN (12.28%) and highest for thermolysin (56.45%). Surprisingly, IN predicts correct sequences for a fraction of MS2 scans obtained with EThcD fragmentation, even though no such scans were included in training of the model, albeit with a lower PSM recall rate (17.93% across all 6 proteases). These results indicate that our models are adept at novel protein sequencing, matching database results coverages while eliminating several steps in the workflows, as the sequencing could be performed directly on the protein level without necessitating prior genomic

information. IN is capable of prediction of peptide sequences of various origins, and has a surprising, however limited, success when predicting sequences from similar fragmentation schemes (Supplementary Fig. 9). This has the potential to drastically speed up novel therapeutics sequencing, by cutting down on sample preparation time, increasing robustness, and decreasing points of failure. Furthermore, by measuring protein and their levels directly, there is considerable promise in assessment of binding efficiency from protein binder abundance.

InstaNovo detects pathogens in human patient wound fluids

Following these results, we investigated how our model would perform in complex samples where the presence of multiple organisms is likely. To investigate this, we utilised wound fluid exudates from human venous leg ulcer patients, analysed in a previous study [4]. These chronic wounds are prone to infection, therefore we suspected that we could detect potential pathogens present in the wound exudates, which would be missed when analysing the raw data with the human proteome alone. In addition, these samples possess plasma-like complexity with a high protein dynamic range, posing another challenge we would like to assess our performance on. Our model achieves 22.24% peptide recall when tested against the database searches. This is the second lowest peptide recall observed across all datasets tested, and we speculate that this is due to the high dynamic range of wound exudate proteins, and experimental noise in MS/MS scans. With IN, we correctly predicted 849 out of 3,727 database PSMs that could be mapped to the human proteome, belonging to 609 unique peptide sequences and 407 proteins. As expected, the protein best mapped is human serum albumin (ALBU_HUMAN, P02768) with 124 PSMs and 24 unique peptides. By expanding our search to the full MS/MS space from the two runs corresponding to two different wound dressings applied to the wound at different timepoints, IN predicts 2,981 novel PSMs at 5% FDR, a 14% PSM and 14.8% unique peptide detection rate increase compared to database search. The resulting predictions map to 1,804 unique peptides and 624 proteins, rivalling database search results. IN+ detected 1,307 novel PSMs at 5% FDR, whilst slightly decreasing the overall peptide recall. In this dataset, we observed several expected correct predictions under the 5% FDR cutoff, with 10,980 matched PSMs to 5,794 peptides from the same human database without any FDR thresholds. We extended albumin mapping to 1,225 PSMs with 254 unique peptides (most semi- or non-tryptic), a 10-fold increase relative to the database search space, and found analogous results in other proteins (Fig. 5a). Notably, these peptides increased albumin sequence coverage from 35.63% in our predictions from the database search space, to 85.22% in the full search space (although falling short of the database search PSMs coverage which was 92.93%). To confirm our hypothesis, we extended our proteome database to include 4 more reference proteomes from pathogens commonly found in wound fluids (*E. Coli, P. aeruginosa, S. aureus* and *Citrobacter sp.* [16–18]). A small fraction of IN at 5% FDR were sequences mapping to

pathogens of interest. Even without FDR thresholds, the expected value of randomly mapped hits to the pathogens with a database of that size would be approximately 40, with probability of false positives at 6-8 residues, different from the results we obtain, indicating that some of these hits are bona fide PSMs. We mapped unique sequences to 5 of *P. aeruginosa*, 23 of *E. Coli*, and 24 of *Citrobacter* sp. proteins, with a significant number of sequences mapping to multiple proteomes (Extended Fig. 5).

Conducting a search with the same database containing the pathogen proteomes, confirmed the presence of the same pathogens in our samples with 80 protein and 130 peptide groups, which demonstrates a robust performance of our models in detecting additional proteins originating from unknown species in biological samples.

InstaNovo identifies additional organisms in complex bacterial communities

Building upon our previous results, we next questioned how IN performs in the field of metaproteomics, i.e. in complex samples where multiple organisms are present. We chose a co-culture of an enrichment reactor for the marine bacterium *Candidatus* "Scalindua brodae", which cannot be grown in isolation as of present, and therefore are not classified as a species yet. We hypothesised that since it is unable to self sustain growth, we would detect other organisms present in the culture. IN achieved a recall of 71.5% at full coverage when evaluated against database search results, the highest observed ostensibly due to low proteome complexity. At 1% FDR, we recorded an 8.77% recall, reaching 58.2% at 5% FDR, indicating a need for post-processing tools that sharpen confidence and widen the distance between true positives and false positive results for use in $<$ 1% FDR searches (Fig. 5b). In addition, IN predicts 3,076 more PSMs in the full search space at 5% FDR, a 33.98% increase compared to database search results. Using IN+ and 5% FDR, we detected an additional 293 novel PSMs from database searches and observed a small increase in the total recall, while predicting 2,192 novel PSMs. In the database search space of 9,053 PSMs satisfying our input criteria, IN correctly predicted 5,402 PSMs mapping to 4,896 unique peptide sequences and 1,460 proteins at 5% FDR. When predicting sequences for all MS/MS scans and the same FDR equivalent of model confidence, we match 6,701 PSMs that map to proteins in our *Candidatus* "Scalindua brodae" database, corresponding to 6,126 unique peptide sequences and 1,531 proteins, of which 3,330 peptide sequences are novel, while extending protein identifications by 26. At the same FDR, we predicted 2,106 high confidence predictions that remained unaccounted for when searching against the *S. brodae* proteome. When checking against a database containing sequences for some of the organisms identified with metagenomics in this culture and reference proteomes of related *S. brodae* species, we identified 88 predictions matching 82 peptides from 74 protein sequences of several additional species (*S. rubra*, *"Candidatus Kuenenia stuttgartiensis"*, *Geobacter sp.*, and *Sulfurovum sp.*), 41 of which are sequences with 9 residues or longer. We examined the

rest of the 1,937 sequences that do not map to any of our databases by comparing them to sequences in genome databases. Using pBLAST and filtering with low expected values (<0.0001) and high identities, we observed potential additional species present in our samples, such as *Phototrophicales bacterium, Candidatus Scalindua arabica, Phycisphaerales bacterium, Bacteroidota bacterium,* and *Gemmatimonadota bacterium* (Fig. 5c). These results illustrate that IN is suitable for metaproteomics applications, where multiple organisms are present in analysed samples, with no prior knowledge about presence of these organisms required.

InstaNovo reveals novel peptides in snake venom and enables comprehensive venomics profiling

Next, we wondered if we could apply our models to samples where limited genomic information is available, and there is a potential for novel sequences to be discovered and new insights to be gained. We therefore selected a dataset that recently described the proteome composition of 26 medically relevant snake venoms from sub-Saharan Africa [19]. We argue that since not all genomes are available, and these proteomes were searched against a pan-snake proteome database, we might observe potential novel sequences unique for some of these species. IN model achieved recall of 18.85% (potentially due to amino acid preference and divergence from training data). IN+ detected an additional 731 correct PSMs from database searches whilst slightly decreasing the overall peptide recall. However, IN expands on the database search results by predicting 5,565 more PSMs in the full search space at a confidence threshold equivalent to 5% FDR, resulting in 1,669 novel peptides and 303 novel protein identifications from the same protein database. Strikingly, these predictions constituted a 54.5% increase in peptide and 46.19% in protein detection rates with our *de novo* sequencing approach, significantly expanding database search results (Fig. 5d). Out of the high confidence predictions in the full search space, we identified 4,113 PSMs that match our protein database, while 2,284 PSMs and 1,117 unique peptide sequences are novel. Using protein Blast and an expected value of < 0.0001 in the top high confidence, long sequence predictions, we could detect matches with high similarity to existing sequences. For example, "SLGGVTTEDCPDGQNLCFK" aligned with isoform 1 sequence of MTLP-2 from *N. kaouthia*, a snake species which was not present in our input dataset. This situation echoes the common occurrence in venom research, where the closest homology match often stems from a different snake species or region and highlights the limitations of relying solely on existing references. Additional matches like "LHSWVECETGECCDQCR" mapped to a snake venom metalloprotease from *E. ocellatus* or "DQGCLPDWSFHEGHCYK" and "DEDCLPDWSSHEGHCYK" mapped to C-type lectins from *Bitis sp.* with a single substitution. Together, these results indicate that these are novel hits with undetected, or not included in the database, search sequences. These can provide valuable insights into novel proteins, isoforms or SNPs in

these samples and assist current development efforts for rationally engineered next-generation antivenoms.

InstaNovo discovers new HLA peptides in immunopeptidome experiments

We next investigated whether our *de novo* sequencing models could be applied to the sequencing of HLA peptides for the analysis of immunopeptidomics experiments. A considerable fraction of our training dataset consisted of HLA peptides, so we were curious to investigate our performance in such evaluation datasets. We chose to evaluate our models with a published interferon induced immunopeptidomics dataset, which was a part of a larger study in high throughput immunopeptidomics [2]. In the complete dataset with a model confidence equivalent to 5% FDR, IN predicted 40,224 PSMs that could be mapped to 8,860 unique peptides and 5,377 proteins in the same human proteome database used in the study, as well as 3,049 peptides in 9,759 PSMs with no matches to the human proteome. Out of the PSMs identified with IN, 28,204 were common with the TD search results, corresponding to a percentage of 28.43%. Remarkably, IN predicts 3,495 novel peptides compared to the TD search, increasing peptide identification rate by 41.53%. IN+ at 5% FDR detected 11,392 more PSMs from the TD search and predicted 12,965 novel PSMs (Fig. 5e). The predicted peptide length formed a distribution centred around 9 residues, with the vast majority (>95%) of predictions being within 8-11 residues. The 9-mer peptides detected with IN showed a motif consistent with MHC bound peptides, exhibiting preferences for certain residues in positions 2 and 9, supporting the model predictions (Fig. 5f). These results illustrate that IN performs well in open searches, is adept in prediction of HLA peptide sequences, and can considerably enhance identification rates in immunopeptidome datasets.

InstaNovo can help detect proteolytic products in terminomics experiments

Lastly, we investigated our model's performance in limited processing or degradomic samples, where proteolytic substrates and their discovery are of interest. We hypothesised that our model would perform well in sequencing of semi- or non-tryptic peptides, since our training dataset contained non-tryptic peptides generated with other proteases, similar to the HLA peptides examined above and results found in other datasets. Proteolytic processing is an ubiquitous PTM in health and disease, degrading or activating various proteins dependent on signalling and stimuli from the environment. We applied our model to a HeLa proteome incubated with GluC or without GluC in triplicate, before preparing them for MS analysis with standard workflows. GluC is a protease cleaving substrate at the C-terminal side of glutamate residues, therefore we sought to detect such peptides with high confidence to evaluate IN's performance protease substrate detection. IN obtained a recall of 68.84%

in this degradomic dataset, when assessed against a semi-tryptic database search for control and GluC treated samples. The improved performance in this dataset, which is also a HeLa proteome, indicates that multiple shots of the same proteome or increased number of PSMs results in better model predictions and accuracy. We mapped 66,000 predicted PSMs with a 5% FDR to the human proteome in the database search space, while this number increased by 13.84% (75,140 predicted PSMs) in the full search space. These predictions corresponded to 22,425 unique peptides in full search space, a 20% increase compared to the database search space. When contrasted with the database search results, IN predicted 4,635 new peptide sequences and improved peptide detection rate by 11.29%, and protein detection rate by 7.11% (Extended Fig. 7a and b). IN+ detected an additional 5,696 correct PSMs from the database search, whilst slightly increasing the total recall. Notably, IN predicted 1,222 new sequences that match the protease profile, i.e. are preceded by glutamate residue in the respective protein sequences these peptides map to (Extended Fig. 7c and d). This reflected a 21.84% increase in putative protease cleavages compared to the database search results. Following this, we questioned whether these cleavages reflected bona fide peptide detections that were missed by database searches. We therefore revisited our samples and interrogated them with targeted proteomics and PRM precursor monitoring. We were able to detect a number of high confidence, semi-tryptic or fully GluC generated peptides, and monitor their fragmentation transitions in both conditions (Fig. 5g). When we matched the IN predicted sequences with the abundance computed with the database search results and performed statistics on them to discern significant changes compared to our controls (two-sided two sample independent t-test, filtering for log2 fold change > 2 and p-value < 0.01), we could obtain a specificity profile with glutamate significantly over-represented at P1 position, just before the cleavage site (Fig. 5h). These results confirm our hypothesis that IN can be applied to the detection of protease substrates at a system-wide scale, and highlight potential applications in degradomics experiments with semi-tryptic or open searches. Similarly, we expect our model to be widely applicable in the detection of truncated proteoforms, exopeptidase processing, and protein degradation.

References

[1] Beslic, D., Tscheuschner, G., Renard, B.Y., Weller, M.G., Muth, T.: Comprehensive evaluation of peptide de novo sequencing tools for monoclonal antibody assembly. Briefings in Bioinformatics **24**(1), 542 (2023)

[2] Chong, C., Marino, F., Pak, H., Racle, J., Daniel, R.T., Müller, M., Gfeller, D., Coukos, G., Bassani-Sternberg, M.: High-throughput and sensitive immunopeptidomics platform reveals profound interferon γ-mediated remodeling of the human leukocyte antigen (hla) ligandome. Molecular & Cellular Proteomics **17**(3), 533–548 (2018)

[3] Nguyen, G.T.T., O'Brien, C., Wouters, Y., Seneci, L., Gallissà-Calzado, A., Campos-Pinto, I., Ahmadi, S., Laustsen, A.H., Ljungars, A.: High-throughput proteomics and in vitro functional characterization of the 26 medically most important elapids and vipers from sub-saharan africa. GigaScience **11**, 121 (2022). https://doi.org/10.1093/gigascience/giac121

[4] Mikosiński, J., Kalogeropoulos, K., Bundgaard, L., Larsen, C.A., Savickas, S., Haack, A.M., Pańczak, K., Rybołowicz, K., Grzela, T., Olszewski, M.: Longitudinal evaluation of biomarkers in wound fluids from venous leg ulcers and split-thickness skin graft donor site wounds treated with a protease-modulating wound dressing. Acta Dermato-Venereologica **102** (2022)

[5] Beslic, D., Tscheuschner, G., Weller, M.G., Renard, B.Y., Muth, T.: Supplementary Data for "Comprehensive evaluation of peptide de novo sequencing tools for monoclonal antibody assembly". Figshare (2022). https://doi.org/10.6084/m9.figshare.21394143.v1. https://doi.org/10.6084/m9.figshare.21394143.v1

[6] Voronov, G., Lightheart, R., Davison, J., Krettler, C.A., Healey, D., Butler, T.: Multi-scale sinusoidal embeddings enable learning on high resolution mass spectrometry data. arXiv preprint arXiv:2207.02980 (2022)

[7] Hoogeboom, E., Nielsen, D., Jaini, P., Forré, P., Welling, M.: Argmax flows and multinomial diffusion: Learning categorical distributions **34**, 12454–12465 (2021)

[8] Yilmaz, M., Fondrie, W.E., Bittremieux, W., Nelson, R., Ananth, V., Oh, S., Noble, W.S.: Sequence-to-sequence translation from mass spectra to peptides with a transformer model. bioRxiv, 2023–0103522621 (2023). https://doi.org/10.1101/2023.01.03.522621

[9] Tran, N.H., Zhang, X., Xin, L., Shan, B., Li, M.: De novo peptide sequencing by deep learning. Proceedings of the National Academy of Sciences **114**(31), 8247–8252 (2017). https://doi.org/10.1073/pnas.1705691114

[10] Zolg, D.P., Wilhelm, M., Schnatbaum, K., Zerweck, J., Knaute, T., Delanghe, B., Bailey, D.J., Gessulat, S., Ehrlich, H.-C., Weininger, M.: Building proteometools based on a complete synthetic human proteome. Nature methods **14**(3), 259–262 (2017)

[11] Pardon, E., Laeremans, T., Triest, S., Rasmussen, S.G.F., Wohlkönig, A., Ruf, A., Muyldermans, S., Hol, W.G.J., Kobilka, B.K., Steyaert, J.: A general protocol for the generation of nanobodies for structural biology. Nature Protocols **9**(33), 674–693 (2014). https://doi.org/10.1038/nprot.2014.039

[12] Ledsgaard, L., Wade, J., Jenkins, T.P., Boddum, K., Oganesyan, I., Harrison, J.A., Villar, P., Leah, R.A., Zenobi, R., Schoffelen, S., Voldborg, B., Ljungars, A., McCafferty, J., Lomonte, B., Gutiérrez, J.M., Laustsen, A.H., Karatt-Vellatt, A.: Discovery and optimization of a broadly-neutralizing human monoclonal antibody against long-chain α-neurotoxins from snakes. Nature Communications **14**(1), 682 (2023). https://doi.org/10.1038/s41467-023-36393-4

[13] Laustsen, A.H., Karatt-Vellatt, A., Masters, E.W., Arias, A.S., Pus, U., Knudsen, C., Oscoz, S., Slavny, P., Griffiths, D.T., Luther, A.M., Leah, R.A., Lindholm, M., Lomonte, B., Gutiérrez, J.M., McCafferty, J.: In vivo neutralization of dendrotoxin-mediated neurotoxicity of black mamba venom by oligoclonal human igg antibodies. Nature Communications **9**(1), 3928 (2018). https://doi.org/10.1038/s41467-018-06086-4

[14] Molina, M.A., Codony-Servat, J., Albanell, J., Rojo, F., Arribas, J., Baselga, J.: Trastuzumab (herceptin), a humanized anti-her2 receptor monoclonal antibody, inhibits basal and activated her2 ectodomain cleavage in breast cancer cells. Cancer research **61**(12), 4744–4749 (2001)

[15] Romond, E.H., Perez, E.A., Bryant, J., Suman, V.J., Geyer Jr, C.E., Davidson, N.E., Tan-Chiu, E., Martino, S., Paik, S., Kaufman, P.A.: Trastuzumab plus adjuvant chemotherapy for operable her2-positive breast cancer. New England journal of medicine **353**(16), 1673–1684 (2005)

[16] Garcia, T.d.F., Borges, E.L., Junho, T.O.d.C., Spira, J.A.O.: Microbiological profile of leg ulcer infections: Review study. Revista Brasileira de Enfermagem **74** (2021)

[17] Cwajda-Białasik, J., Mościcka, P., Jawień, A., Szewczyk, M.T.: Microbiological status of venous leg ulcers and its predictors: a single-center cross-sectional study. International Journal of Environmental Research and Public Health **18**(24), 12965 (2021)

[18] Gjødsbøl, K., Christensen, J.J., Karlsmark, T., Jørgensen, B., Klein, B.M., Krogfelt, K.A.: Multiple bacterial species reside in chronic wounds: a longitudinal study. International wound journal **3**(3), 225–231 (2006)

[19] Nguyen, G.T.T., O'Brien, C., Wouters, Y., Seneci, L., Gallissà-Calzado, A., Campos-Pinto, I., Ahmadi, S., Laustsen, A.H., Ljungars, A.: High-throughput proteomics and in vitro functional characterization of the 26 medically most important elapids and vipers from sub-saharan africa. GigaScience **11**, 121 (2022). https://doi.org/10.1093/gigascience/giac121
